# The illusion of moral decline

**DOI:** 10.1038/s41586-023-06137-x

**Published:** 2023-06-07

**Authors:** Adam M. Mastroianni, Daniel T. Gilbert

**Affiliations:** 1 Columbia University, New York, NY USA; 2 Harvard University, Cambridge, MA USA

**Keywords:** Human behaviour, Psychology

## Abstract

Anecdotal evidence indicates that people believe that morality is declining^1,2^. In a series of studies using both archival and original data (*n* = 12,492,983), we show that people in at least 60 nations around the world believe that morality is declining, that they have believed this for at least 70 years and that they attribute this decline both to the decreasing morality of individuals as they age and to the decreasing morality of successive generations. Next, we show that people’s reports of the morality of their contemporaries have not declined over time, suggesting that the perception of moral decline is an illusion. Finally, we show how a simple mechanism based on two well-established psychological phenomena (biased exposure to information and biased memory for information) can produce an illusion of moral decline, and we report studies that confirm two of its predictions about the circumstances under which the perception of moral decline is attenuated, eliminated or reversed (that is, when respondents are asked about the morality of people they know well or people who lived before the respondent was born). Together, our studies show that the perception of moral decline is pervasive, perdurable, unfounded and easily produced. This illusion has implications for research on the misallocation of scarce resources^3^, the underuse of social support^4^ and social influence^5^.

## Abstract (web summary)

We show that the perception of moral decline is pervasive, perdurable, unfounded and easily produced, and suggest that this illusion has implications for research on the misallocation of scarce resources, the underuse of social support and social influence.

## Main

The social fabric appears to be unravelling: civility seems like an old-fashioned habit, honesty like an optional exercise and trust like the relic of another time. Some observers^6^ claim that “the process of our moral decline” began with the “sinking of the foundations of morality” and proceeded to “the final collapse of the whole edifice”, which brought us “finally to the dark dawning of our modern day, in which we can neither bear our immoralities nor face the remedies needed to cure them”. But as apt as this description of our times may seem, it was written more than 2,000 years ago by the historian Livy, who was bemoaning the declining morality of his fellow Roman citizens. From ancient to modern times, social observers have often lamented the ugly turns their societies have taken, and have often suggested that a recent decline in morality—in kindness, honesty and basic human decency—was among the causes^2,7^.

Why have so many different people in so many different times and places been convinced that their fellow citizens are now less moral than they once were? One possibility is that morality has, in fact, been declining worldwide for millennia—declining so steadily and so precipitously that people in every era have been able to observe that decline in the brief span of a human lifetime. The other possibility is that the perception of moral decline is a psychological illusion to which people all over the world and throughout history have been susceptible. We provide evidence for the latter possibility. First, we show that people in at least 60 nations do indeed believe that morality is declining, and that they have believed this for at least 70 years. Second, we show that people attribute this decline both to the decreasing morality of individuals as they age and to the decreasing morality of successive generations. Third, we show that people’s reports of the current morality of their contemporaries have not declined over time, which strongly suggests that the perception of moral decline is an illusion. Fourth and finally, we describe tests of a simple psychological mechanism that can produce the illusion of moral decline and can predict some of the circumstances under which it will be attenuated, eliminated or reversed (for example, when respondents are asked about the morality of people they know well or people who lived before the respondent was born).

## Do people perceive moral decline?

Morality refers primarily to people’s treatment of each other^8^, which ranges from the altruistic^9^ to the barbaric^10^. But like most social observers, Livy was not remarking on the moral extremes—on the rare heroic deed or occasional heinous crime that few people ever perform or experience. Rather, he was remarking on the ways in which ordinary people behave in their daily lives. Do modern people, like Livy, believe that their contemporaries are less honest and kind than they used to be? Do they think their neighbours are less generous and less helpful, that their co-workers are more likely to treat each other disrespectfully and betray each other’s trust? Survey researchers have been asking people about their perceptions of changes in these everyday moral qualities since at least 1949, but the full corpus of relevant survey data has never been systematically assembled and analysed. We began by doing that.

In study 1, we searched the databases of major survey research providers (using search terms shown in the Supplementary Information) and found 177 survey items that asked representative samples of a total of 220,772 US Americans if and how they thought other people’s morality had changed over time (Supplementary Table 1). These items were administered over a 70-year span from 1949 to 2019. Typical items included: “Do you think that over the last few decades our society has become less honest and ethical in its behavior, more honest and ethical, or has there been no change in the extent to which people behave honestly and ethically?” and “Right now, do you think the state of moral values in this country as a whole is getting better or getting worse?” (further methodological details can be found in the concluding method section as well as in the Supplementary Information). On 84.18% of the items, the majority of participants reported that morality had declined. A linear model indicated that the proportion of participants who reported moral decline was not significantly influenced by the year in which the survey was administered, *b* = 0.07, 95% confidence interval (CI) = [−0.11, 0.24], *t*(175) = 0.77, *P* = 0.45, adjusted *R*^2^ = −0.002, and the same model fit in a Bayesian framework indicated strong evidence of no effect (Bayes Factor of 0.04), which is to say that US Americans have been reporting moral decline at the same rate for as long as researchers have been asking them about it. (These and all tests we report are two-tailed).

Two more findings were noteworthy. First, participants in study 1 were more likely to perceive moral decline when they were asked about longer periods of time (for example, “the last decade”) than about shorter periods of time (“the last year”), *b* = 0.57, 95% CI = [0.09, 1.05], *t*(43) = 2.42, *P* = 0.02, adjusted *R*^2^ = 0.10, which is precisely what one would expect if participants believed that morality has been declining continuously. Second, participants reported increases in morality when asked about a few specific issues on which social progress has clearly been made: for example, 59% of participants reported improved treatment of African Americans, 51% reported improved treatment of people with physical disabilities and 50% reported improved treatment of gay people. The fact that participants calculated moral decline cumulatively across time periods and acknowledged special exceptions to the general trend suggests that they were reporting well-considered beliefs, and not merely expressing some vague sense of despair about humanity. Indeed, in the Supplementary Information, we report an extra study (Supplementary study 3) showing that the perception of moral decline persists even when people are incentivized to respond accurately.

**Fig. 1:**
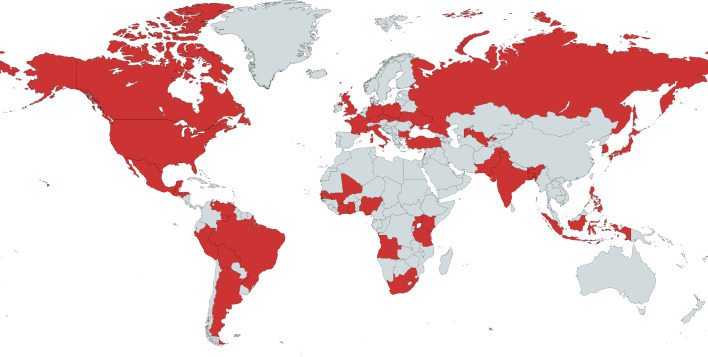
Countries surveyed by Pew in 2002 or 2006. In every country surveyed by Pew in 2002 or 2006 (shown in red), the majority of participants reported that moral decline was at least a “moderately big problem”. Map created with MapChart.

The perception of moral decline was not unique to US Americans. We resampled the databases of major survey research providers and found 58 survey items that asked a total of 354,120 participants in 59 nations other than the United States if and how they thought other people’s morality had changed over time (Supplementary Table 2). These items were administered over a 13-year span from 1996 to 2007. An analysis of these items showed that on 86.21% of the items, the majority of non-US participants reported that morality had declined. Indeed, the Pew Research Center surveyed citizens of 40 nations in 2002 (ref. ^11^) and 2006 (ref. ^12^) and, as Fig. 1 shows, in every one of those nations, the majority of participants reported that moral decline was at least a “moderately big problem”.

The survey items we analysed in study 1 (Supplementary Tables 1 and 2) used a wide range of question formats to ask participants over a wide range of decades about moral decline across a wide range of time periods, and they converged on a single conclusion: people all over the world believe that morality has declined, and they have believed this for as long as researchers have been asking them about it. Archival data are uniquely able to tell us how people in the past thought and felt, but they have limits. Some of the items we analysed asked participants for their perceptions of changes in “moral values” without specifying what those values were (for example, “Right now, do you think the state of moral values in this country as a whole is getting better or getting worse?”), some failed to specify the time in the past to which the present was to be compared (for example, “Compared to the past, are people today more or less friendly toward their neighbors?”) and some contained ambiguous wording that was not optimal for extracting accurate measures of people’s perceptions of moral decline (“Considering just the moral climate of the country today, do you feel things in this country are generally going in the right direction or do you feel things have pretty seriously gotten off on the wrong track?”). In addition, all items asked participants questions about the presence or absence of moral decline rather than asking them to rate the level of morality of people in both the present and the past, which allowed us to compute the proportion of participants who perceived moral decline but not how much decline they perceived. We addressed these and other limitations of the archival data by conducting three original studies.

**Fig. 2:**
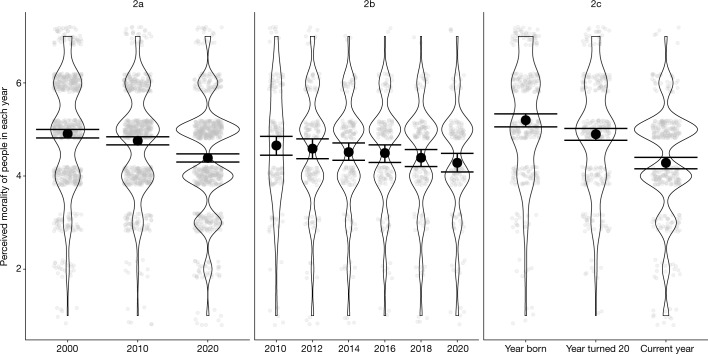
Results of studies 2a–c. The panels show the results of studies 2a (left panel), 2b (middle panel) and 2c (right panel). Opaque points represent means. Transparent points represent individual observations jittered for legibility. Error bars represent 95% CIs. Study 2a *n* = 698, study 2b *n* = 148 and study 2c *n* = 347.

In studies 2a–c, we asked samples of US Americans to rate how “kind, honest, nice, and good” people were in 2020 (the year the studies were conducted), as well as in various other years that differed by study. Methodological details can be found in the concluding method section and in the Supplementary Information. As Fig. 2 shows, participants in study 2a (*n* = 698 respondents on Prolific), study 2b (*n* = 185 respondents on Amazon Mechanical Turk) and study 2c (*n* = 347 respondents on Amazon Mechanical Turk) perceived moral decline. Specifically, in study 2a, participants rated people as less kind, honest, nice and good in 2020 (mean (*M*) = 4.39) than in 2010 (*M* = 4.76, *b* = −0.37, 95% CI = [−0.46, −0.28], *t*(1394) = −9.38, *P* < 0.001, Cohen’s *d* = −0.50), or in 2000 (*M* = 4.91, *b* = −0.52, 95% CI = [−0.62, −0.43], *t*(1394) = −13.23, *P* < 0.001, *d* = −0.71), and as less kind, honest, nice and good in 2010 than in 2000, *b* = −0.15, 95% CI = [−0.25, −0.06], *t*(1394) = −3.85, *P* < 0.001, *d* = −0.21. We also conducted two direct replications of study 2a (one of which was preregistered; Supplementary Information), which produced the same results.

In study 2b, participants rated people as less kind, honest, nice and good in 2020 (*M* = 4.28) than in 2016 (*M* = 4.49, *b* = −0.21, 95% CI = [−0.43, 0.006], *t*(735) = −2.87, *P* = 0.047, *d* = −0.33), in 2014 (*M* = 4.51, *b* = −0.23, 95% CI = [−0.45, −0.01], *t*(735) = −3.14; *P* = 0.02, *d* = −0.37), in 2012: *M* = 4.59, *b* = −0.30, 95% CI = [−0.52, −0.09], *t*(735) = −4.16, *P* < 0.001, *d* = −0.48) and in 2010 *M* = 4.66, *b* = −0.37, 95% CI = [−0.59, −0.16], *t*(735) = −5.08, *P* < 0.001, *d* = −0.59). Participants in study 2b also rated people in 2018 as less kind, honest, nice and good than in people in 2010 (*b* = −0.26, 95% CI = [−0.48, −0.05], *t*(735) = −3.61, *P* = 0.004, *d* = −0.32). No other comparisons were significant (all *P* > 0.05).

In study 2c, participants rated people as less kind, honest, nice and good in 2020 (*M* = 4.28) than in the year the participant turned 20 years old (*M* = 4.89, *b* = −0.61, 95% CI = [−0.77, −0.45], *t*(675) = −9.21, *P* < 0.001, *d* = −0.72) and in the year the participant was born (*M* = 5.20, *b* = −0.92, 95% CI = [−1.07, −0.76], *t*(667) = −14.27, *P* < 0.001, *d* = −1.08). Participants also rated people as less kind, honest, nice and good in the year the participant turned 20 years old than in the year the participant was born, *b* = −0.31, 95% CI = [−0.47, −0.15], *t*(675) = −4.70, *P* < 0.001, *d* = −0.37.

In studies 2a–c, we also examined the effects of age, gender, race, education (1 = did not finish high school; 6 = graduate degree), political ideology (−2 = very liberal; 2 = very conservative) and parental status (0 = not parent; 1 = parent) on perceptions of moral decline using an exploratory linear regression. In study 2a, more conservative participants perceived more decline, *b* = −0.18, 95% CI = [−0.27, −0.09], *t*(684) = −4.03, *P* < 0.001. No other effects in study 2a were significant (all *P* > 0.05). An additional exploratory one-sample *t*-test indicated that, although more conservative participants perceived more moral decline than did more liberal participants, more liberal participants perceived moral decline as well, *M* = −0.35, 95% CI = [−0.47, −0.23], *t*(398) = −4.96, *d* = 0.28, *P* < 0.001, *d* = 0.28. In study 2b, no other effects were significant (all *P* > 0.05). In study 2c, more conservative participants perceived more moral decline than did more liberal participants, *b* = −0.15, 95% CI = [−0.28, −0.12], *t*(329) = −2.15, *P* = 0.03, but more liberal participants perceived moral decline as well, *M* = −0.80, 95% CI = [−1.02, −0.58], *t*(159) = −7.23, *P* < 0.001, *d* = 0.57.

In study 2c, older participants perceived more moral decline than did younger participants, *b* = −0.02, 95% CI = [−0.03, −0.004], *t*(329) = −2.61, *P* = 0.01. No other effects were significant (all *P* > 0.05). We further investigated the effect of age in study 2c by creating two moral decline scores: specifically, (1) we subtracted participants’ ratings of people in the year the participant was 20 years old from their ratings of people in 2020 and (2) we subtracted participants’ ratings of people in the year the participant was born from their ratings of people in 2020. An exploratory linear model indicated that older participants perceived more moral decline than did younger participants both compared to the year in which they turned 20 years old: *b* = −0.02, *t*(320) = −3.30, 95% CI = [−0.03, −0.007], *P* < 0.001, and compared to the year in which they were born: *b* = −0.02, *t*(345) = −3.77, 95% CI = [−0.04, −0.01], *P* < 0.001. Did older participants perceive more moral decline simply because they were considering longer periods of time? Yes. We created a measure of the annual rate of moral decline by subtracting the participants’ ratings of people in the year the participant was born from their rating of people in 2020, and then dividing that value by the participant’s age. We then fit an exploratory linear model with the perceived annual rate of moral decline as the outcome and age as a predictor. The main effect of age was not significant, *b* = −0.0002, 95% CI = [−0.0005, 0.0002], *t*(345) = −1.05, *P* = 0.29, and refitting the same model in a Bayesian framework provided strong evidence that perceived moral decline per year did not differ by age (100% of high-density interval (HDI) in the region of practical equivalence (ROPE)). In other words, younger and older participants did not report different annual rates of moral decline, which is to say that they reported different total amounts of moral decline only because they were reporting on moral decline across different numbers of years.

## To what do people attribute moral decline?

People clearly perceive moral decline, but to what do they attribute it? There are two possibilities. The average morality of a population may decline between two points in time (*T*_1_ and *T*_2_) because (1) individuals who are moral at *T*_1_ are less moral when they reach *T*_2_ (a phenomenon we refer to as ‘personal change’), and/or (2) older people who were alive at *T*_1_ but who died before *T*_2_ are more moral than younger people who were alive at *T*_2_ but who were not yet born (or who were not yet adults and therefore not sampled) at *T*_1_ (a phenomenon we refer to as ‘interpersonal replacement’). When the average morality of a population declines over very short time periods (for example, a day), the decline is probably the result of personal change (because very few people who were measured at *T*_1_ were not also measured at *T*_2_), and when the average morality of a population declines over very long time periods (for example, 200 years), the decline is necessarily the result of interpersonal replacement (because no human being lives for 200 years and therefore no one who was measured at *T*_1_ was also measured at *T*_2_).

So, what about moral decline over the intermediate time periods that participants in studies 2a–c were asked about? To which of these sources—personal change or interpersonal replacement—do people attribute such decline? In study 3 (*n* = 319 respondents on Amazon Mechanical Turk), we asked a sample of US Americans to rate how “kind, honest, nice, and good” people were in 2020 (the year the study was conducted) and in 2005. Methodological details can be found in the Methods and in the Supplementary Information. Next, participants rated the morality of two exclusive subsets of this population. The first subset was people who were living adults in both 2005 and 2020. The difference between these ratings was a measure of participants’ perceptions of personal change between the 2 years. The second subset was people who were living adults in either 2005 or 2020, but not in both years. The difference between these ratings was a measure of participants’ perceptions of interpersonal replacement between the 2 years.

A paired samples *t*-test indicated that participants perceived moral decline, rating people as less kind, honest, nice and good in 2020 (*M* = 4.35) than in 2005 (*M* = 4.89), *t*(318) = −9.88, 95% CI = [−0.65, −0.44], *d* = 0.55, *P* < 0.001. To determine whether participants attributed this moral decline to personal change and/or to interpersonal replacement, we used linear regression to determine whether and how well participants’ perceptions of personal change and of interpersonal replacement predicted their perceptions of moral decline. Both measures significantly predicted participants’ perceptions of moral decline (personal change *b* = 0.50, 95% CI = [0.40, 0.59], *t*(316) = 9.96, *P* < 0.001; interpersonal replacement *b* = 0.17, *t*(316) = 6.52, 95% CI = [0.12, 0.22], *P* < 0.001; adjusted *R*^2^ = 0.36). We refit the model to include age, gender, race, political ideology, education and parental status as covariates, and the effects of personal change and interpersonal replacement both remained significant (personal change *b* = 0.50, 95% CI = [0.40, 0.60], *t*(304) = 9.87, *P* < 0.001; interpersonal replacement *b* = 0.18, 95% CI = [0.13, 0.23], *t*(304) = 6.71, *P* < 0.001; adjusted *R*^2^ = 0.38).

In short, participants in study 3 believed that morality had declined on average over a 15-year period, and they attributed that decline both to the decreasing morality of individuals over time and to the decreasing morality of successive generations^13,14^. The fact that people attribute moral decline to both sources may help explain why their perceptions of moral decline are so robust, appearing in study 3, in the archival data of study 1 and in the original data collected for studies 2a–c.

## Is morality declining?

People believe that morality is declining. Is it? Societies keep (or at least leave) reasonably good records of extremely immoral behaviour such as slaughter and conquest, slavery and subjugation or murder and rape, and careful analyses of those historical records strongly suggest that these objective indicators of immorality have decreased significantly over the last few centuries^15,16^. On average, modern humans treat each other far better than their forebears ever did—which is not what one would expect if honesty, kindness, niceness and goodness had been decreasing steadily, year after year, for millennia. Although there are no similarly objective historical records of everyday morality—of how often people offer their seats to an elderly person, give directions to a lost tourist or help their neighbour fix a fence—there are subjective measures of such things.

Recall that in study 1, we examined people’s reports of moral change, which were obtained when survey researchers asked people to mentally compare the morality of people in the present to the morality of people at some point in the past and then report the direction of the difference. But, for decades, survey researchers have also been asking people to report directly on the moral values, traits and behaviours of themselves and their contemporaries in the present: “Were you treated with respect all day yesterday?” or “Would you say that most of the time people try to be helpful, or that they are mostly just looking out for themselves?” or “During the past 12 months, how often have you carried a stranger’s belongings, like groceries, a suitcase, or shopping bag?” (Supplementary Tables 3 and 4). If, as people all over the world claim, morality has been declining steadily and precipitously for decades, then people’s reports of current morality should also have declined over the years. Have they?

In study 4, we searched the databases of major survey research providers (using search terms listed in the Supplementary Information) and found 107 items that were administered to 4,483,136 people across a 55-year span from 1965 to 2020, and that (1) asked participants to report on some aspect of current morality and (2) were administered at least twice, at times that were at least 10 years apart (Supplementary Table 3 shows the items). To determine whether people’s reports of the current morality of their contemporaries changed over time, we fit a linear model for each survey. The year of each survey was always entered as a predictor, and the outcome was always the average perception of current morality. Because these surveys generally had large samples—some with hundreds of thousands of participants—the significance of *P* values is not very meaningful, so we used *R*^2^ values as a measure of effect size. To shed further light on the size of these effects, we also fit analogous models in a Bayesian framework.

The results of both analyses were clear: people’s reports of the current morality of their contemporaries were stable over time. On average, the year in which the survey was conducted explained less than 0.3% of the variance in responses, and in almost all cases it explained less than 1% (Supplementary Table 4). This result was confirmed by Bayesian analysis, which showed that 100% of the HDI was within the ROPE in all but one case, indicating that any changes over time were negligible at best. We repeated these analyses for data collected from non-US samples (33 samples, *n* = 7,432,736) and found similar results: on average, the year in which the survey was conducted explained less than 0.2% of the variance in responses (all items and results for the non-US sample are listed in Supplementary Table 4). In short, studies 1–3 showed that when people are explicitly asked to assess moral change, they claim that morality has declined, but study 4 shows that when people are asked to assess the current morality of their contemporaries, their assessments do not change over time.

Could this be because words can change meaning over time? If residents of Los Angeles in both 1942 and 2022 described traffic as ‘heavy’, it would be a mistake to conclude that traffic had not actually increased. Words such as ‘heavy’ and ‘moral’ are inherently ambiguous, and if people adapt to changes in traffic or morality, then people in different decades may use the same ambiguous word to describe very different states of affairs. This is unlikely to be the case in study 4 because in addition to including a few items that measured traits and values with ambiguous terms such as ‘morality’, the dataset (Supplementary Tables 3 and 4) mainly contained items that measured specific and relatively unambiguous moral behaviours, such as “Within the past 12 months, have you been assaulted or mugged?” or “During the past 12 months, have you let a stranger go ahead of you in line?” Answers to specific and unambiguous questions such as these did not change over time. It seems rather improbable that people were less likely to allow strangers into a line in 2020 than in 2010, but that somehow in that 10-year span, the meaning of words such as ‘stranger’ and ‘line’ had changed in ways that masked that objective decline in kindness.

The subjective measures we analysed are not definitive, of course, but they strongly suggest that the widespread perception of moral decline is an illusion. Moreover, studies that use the rare objective measure of changes in everyday moral behaviour suggest the same thing. For instance, Yuan et al.^17^ showed that rates of cooperation in the Prisoner’s Dilemma game have increased significantly between 1956 and 2017, and in the Supplementary Information, we report the results of a study (Supplementary study 3) showing that most people mistakenly believe that such cooperation has declined.

## Why do people perceive moral decline?

The results of studies 1–3 suggest that people believe that morality has declined, and the results of study 4 suggest that this belief is illusory. If morality has not declined, then why do people think it has? Although there are surely many good answers to this question, we suggest that one of them has to do with the fact that when two well-established psychological phenomena work in tandem, they can produce an illusion of moral decline. First, numerous studies have shown that human beings are especially likely to seek and attend to negative information about others^18–20^, and mass media indulge this tendency with a disproportionate focus on people behaving badly^21^. As such, people may encounter more negative information than positive information about the morality of ‘people in general’, and this ‘biased exposure effect’ may help explain why people believe that current morality is relatively low. Second, numerous studies have shown that when people recall positive and negative events from the past, the negative events are more likely to be forgotten^22^, more likely to be misremembered as their opposite^23,24^ and more likely to have lost their emotional impact^25^. This ‘biased memory effect’ may help explain why people believe that past morality was relatively high. Working together, these two phenomena can produce an illusion of moral decline. Specifically, biased exposure to information about current morality may make the present seem like a moral wasteland, biased memory for information about past morality may make the past seem like a moral wonderland and when people in a wasteland remember being in a wonderland, they may naturally conclude that the landscape has changed.

This ‘biased exposure and memory’ (BEAM) mechanism comports well with the results of the studies we have described, but it also makes at least two testable predictions. Specifically, the BEAM mechanism predicts that the illusion of moral decline should be attenuated, eliminated or even reversed when (1) people are exposed to a disproportionate amount of positive rather than negative information about the moral behaviour of others, as they are with their families, friends and associates, and (2) when people are asked about times for which they have little or no information in memory, such as in the years before they were born. In the Supplementary Information, we provide a mathematical model of the BEAM mechanism and show how the model makes these two predictions, which we tested in studies 5a and 5b.

Study 5a (*n* = 283 respondents on Amazon Mechanical Turk) tested the hypothesis that the illusion of moral decline is attenuated, eliminated or reversed when participants are asked to rate people in their personal worlds rather than people in general. As described in the Methods (and Supplementary Information), we began by measuring participants’ perceptions of (1) overall moral decline; (2) personal change among people in general and (3) interpersonal replacement among people in general. Then we measured participants’ perceptions of (4) personal change among people in their personal worlds and (5) interpersonal replacement among people in their personal worlds. We explained that the phrase ‘personal worlds’ referred to “all the people with whom you currently interact, in person or otherwise, in your everyday life. This probably includes friends, family members, coworkers, classmates, neighbors, etc.”.

**Fig. 3:**
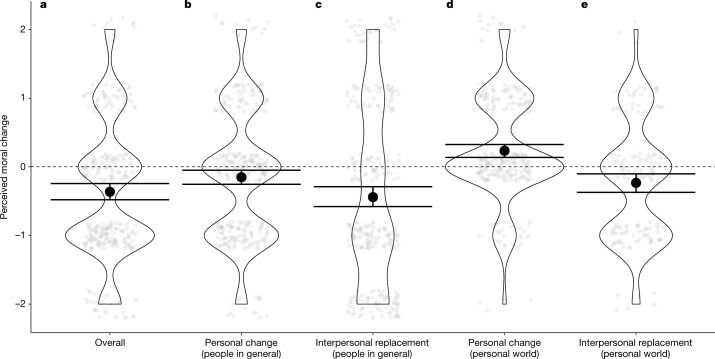
Results of study 5a. **a**–**e**, From left to right, this figure shows the perceived difference in morality of (**a**) people in general in 2005 and people in general in 2020 (overall) (**b**), people in general who were sampled both in 2005 and 2020 (personal change among people in general) (**c**), people in general who were sampled in 2005 or 2020 but not in both years (interpersonal replacement among people in general) (**d**), people in the participant’s personal world who were sampled both in 2005 and 2020 (personal change among people in personal world) and (**e**) people in the participant’s personal world who were sampled in 2005 or 2020 but not in both years (interpersonal replacement among people in participant’s personal world). Opaque points represent means. Transparent points represent individual observations jittered for legibility. Error bars represent 95% CIs. *n* = 283.

We used one-sample *t*-tests to determine whether each of the measures described above differed significantly from zero. First, participants on average perceived moral decline: they believed that people in general were not as kind, honest, nice and good in 2020 as they were in 2005 (*M* = −0.36), *t*(282) = −6.04, 95% CI = [−0.48, −0.25], *d* = 0.36, *P* < 0.001. Second, participants believed that individuals in 2020 were not as kind, honest, nice and good as those same individuals had been in 2005, *M* = −0.15, *t*(282) = −2.67, 95% CI = [−0.26, −0.04], *d* = 0.16, *P* = 0.008, and that younger people in 2020 were not as kind, honest, nice and good as older people were in 2005, *M* = −0.44, *t*(282) = −5.82, 95% CI = [−0.59, −0.29], *d* = 0.35, *P* < 0.001. In other words, as in study 3, participants believed that morality had declined among individuals and between successive generations. These results are illustrated in Fig. 3.

Did participants believe the same things about people in their personal worlds? No. First, participants believed that the individuals who were in their personal worlds in both 2005 and 2020 had shown moral improvement over that period rather than moral decline, *M* = 0.23, *t*(255) = 4.86, 95% CI = [0.14, 0.33], *d* = 0.30, *P* < 0.001. Second, although participants believed that the younger people who were in their personal worlds in 2020 (but not in 2005) were not as kind, honest, nice and good as the older people who were in their personal worlds in 2005 (but not in 2020), *M* = −0.23, *t*(144) = −3.23, 95% CI = [−0.38, −0.09], *d* = 0.27, *P* = 0.002, this difference was smaller among people in their personal worlds than it was among people in general, *t*(144) = −2.56, 95% CI = [−0.40, −0.05], *d* = 0.18, *P* = 0.01.

To investigate the effects of demographic variables on the perception of moral decline, we fit the same exploratory model used in studies 2a–c. The outcome variable was participants’ perceptions of moral decline between 2005 and 2020. Older participants perceived more moral decline than did younger participants, *b* = −0.02, 95% CI = [−0.03, −0.005], *t*(272) = −2.93, *P* = 0.004, and non-parents perceived more moral decline than did parents, *b* = 0.29, 95% CI = [0.02, 0.56], *t*(271) = 2.12, *P* = 0.03, adjusted *R*^2^ = 0.07. No other effects were significant (all *P* > 0.05).

In short, participants in study 5a believed that morality had declined among people in general, but this effect was reversed (in the case of personal change) or attenuated (in the case of interpersonal replacement) among the people they personally knew. We hasten to note that there are surely many reasons why people might think differently about people in their personal worlds than about people in general and that the BEAM mechanism is, at best, just one.

**Fig. 4:**
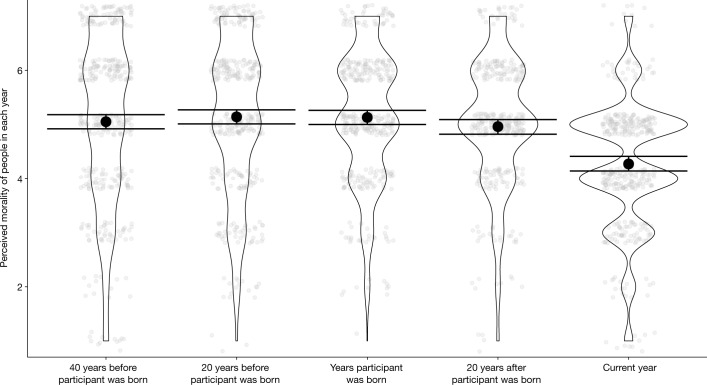
Results of Study 5b. The figure shows the perceived morality of people in various years. Opaque points represent means. Transparent points represent individual observations jittered for legibility. Error bars represent 95% CIs. *n* = 387.

Study 5b (*n* = 387 respondents on Amazon Mechanical Turk) tested the hypothesis that the illusion of moral decline is attenuated, eliminated or reversed when participants are asked to rate the morality of people in general in the years before the participant was born. Participants rated how kind, honest, nice and good people in general are or were at four points in time: in the current year (which was 2021), 20 years after the participant was born, the year the participant was born, 20 years before the participant was born and 40 years before the participant was born. We fit the same model and planned contrasts used in studies 2a–c. As in our previous studies, participants perceived moral decline among people in general in the years after the participant was born. Specifically, participants believed that people in general were (1) less kind, honest, nice and good in 2021 (*M* = 4.27) than they were in the year the participant was 20 years old (*M* = 4.96), *b* = –0.68, 95% CI = [–0.85, –0.51], *t*(1513) = –10.07, *P* < 0.001, *d* = −0.75 and (2) less kind, honest, nice and good in the year the participant was 20 years old than they were in the year the participant was born (*M* = 5.13), *b* = –0.18, 95% CI [–0.35, –0.01], *t*(1513) = –2.60, *P* = 0.03, *d* = −0.19. However, there was no evidence to suggest that participants perceived moral decline in the years before they were born. Specifically, there was no evidence that participants believed that people in general were (1) any more or less kind, honest, nice and good in the year the participant was born (*M* = 5.13) than they were 20 years before the participant was born (*M* = 5.14), *b* = –0.01, 95% CI = [–0.17, 0.15], *t*(1506) = –0.16, *P* = 0.87, *d* = −0.01 and (2) any more or less kind, honest, nice and good 20 years before the participant was born (*M* = 5.14) than they were 40 years before the participant was born (*M* = 5.05), *b* = 0.09, 95% CI = [−0.24, 0.04], *t*(1506) = −1.42, *P* = 0.31, *d* = 0.10. Equivalence tests using the ‘parameters’ package in R^26^ indicated that there was insufficient evidence to conclude that participants’ ratings for 40 years before their birth and 20 years before their birth were equivalent (91.74% of HDI in ROPE, *P* = 0.09; if anything, participants perceived moral improvement between these years), but that there was sufficient evidence to conclude that participants’ ratings for 20 years before their birth and the year of their birth were equivalent (100% of HDI in ROPE, *P* = 0.003). In short, participants believed that moral decline began at about roughly the same time they appeared on Earth. These results are illustrated in Fig. 4.

To investigate the effects of demographic variables on the perception of moral decline, we fit the same exploratory model used in studies 2a–c. The outcome variable was perceived moral decline between the year of the participant’s birth and 2021. More conservative participants perceived more moral decline than did more liberal participants, *b* = −0.33, 95% CI = [−0.45, −0.22], *t*(374) = −5.81, *P* < 0.001, but a one-sample *t*-test indicated that more liberal participants perceived moral decline as well, *b* = −0.46, 95% CI = [−0.64, −0.28], *t*(196) = −5.11, *P* < 0.001, *d* = 0.36. Although older participants perceived more moral decline than did younger participants, *b* = −0.01, 95% CI = [−0.02, −0.003], *t*(374) = −2.60, *P* = 0.009, this was because older participants were perceiving moral decline over a longer period of time. Indeed, the same analysis used in study 2c indicated no evidence that older and younger participants perceived different annual rates of moral decline between the year they were born and 2021, *b* = 0.000005, 95% CI = [−0.0003, 0.0003], *t*(385) = 0.04, *P* = 0.97, consistent with the results of study 2c.

Studies 5a and 5b show that when participants were asked to assess the morality of people about whom they had mainly positive information in memory (that is, people in their personal worlds) or about whom they had little or no information in memory (that is, people who lived before the participants were born), the perception of moral decline was attenuated, eliminated or reversed, just as the BEAM mechanism predicts. The illusion of moral decline is a robust phenomenon that surely has several causes, and no one can say which of them produced the illusion that our studies have documented. Studies 5a and 5b do not directly implicate the BEAM mechanism in that production but they do make it a viable candidate for future research.

## Discussion

Participants in the foregoing studies believed that morality has declined, and they believed this in every decade and in every nation we studied. They believed the decline began somewhere around the time they were born, regardless of when that was, and they believed it continues to this day. They believed the decline was a result both of individuals becoming less moral as they move through time and of the replacement of more moral people by less moral people. And they believed that the people they personally know and the people who lived before they did are exceptions to this rule. About all these things, they were almost certainly mistaken. One reason they may have held these mistaken beliefs is that they may typically have encountered more negative than positive information about the morality of contemporaries whom they did not personally know, and the negative information may have faded more quickly from memory or lost its emotional impact more quickly than the positive information did, leading them to believe that people today are not as kind, nice, honest or good as once upon a time they were.

Like all studies, ours have limitations. For example, studies 1 and 4 made use of archival data that were not collected for the purposes to which we put them and that were therefore less than ideal. For example, some of the items we analysed asked participants for their perceptions of changes in ‘moral values’ without specifying what those values were, some failed to specify the time in the past to which the present was to be compared, and some contained ambiguous wording that was not optimal for extracting accurate measures of people’s perceptions of moral decline. Moreover, all the items asked participants about the presence or absence of moral decline rather than asking them to rate the level of morality of people in both the present and the past. These limitations were addressed by studies 2a–c, but these studies had limitations of their own (for example, all participants were from the United States). And although studies 5a–b demonstrated the viability of the BEAM mechanism, they do not tell us whether it was the cause of the illusion of moral decline that our other studies documented.

With that said, the illusion of moral decline seems to be a robust phenomenon that may have troubling consequences. For example, in 2015, 76% of US Americans agreed that “addressing the moral breakdown of the country” should be a high priority for their government^27^. The United States faces many well-documented problems, from climate change and terrorism to racial injustice and economic inequality—and yet, most US Americans believe their government should devote scarce resources to reversing an imaginary trend. The belief that everyday morality is on the wane may also affect people’s interpersonal behaviour. For example, research shows that people are reluctant to seek the aid and comfort of those whom they do not know because they underestimate how willingly those people would provide it^4,28,29^. The illusion of moral decline may be one of the reasons people do not depend as much as they might on the kindness of strangers—an act that might well ameliorate the illusion itself. The illusion of moral decline may also leave people dangerously susceptible to manipulation by bad actors. Research shows that people are especially influenced by ‘dynamic norms’, which are perceived changes in customary ways of behaving^5^. If low morality is a cause for concern, then declining morality may be a veritable call to arms, and leaders who promise to halt that illusory slide—to “make America great again”, as it were—may have outsized appeal. Our studies indicate that the perception of moral decline is pervasive, perdurable, unfounded and easily produced. Achieving a better understanding of this phenomenon would seem a timely task.

## Methods

### Study 1

In study 1, we conducted keyword-term searches of the Roper Center for Public Opinion iPoll Database, and manually searched the databases of the General Social Survey, Pew Research Center, Gallup, the American National Election Studies, the World Values Survey, the European Social Survey and the European Values Survey to locate survey items that asked participants if and how they thought other people’s morality had changed over time. In our analyses, we included all surveys that (1) used a representative sample of US American participants, and (2) explicitly asked participants about their perceptions of changes in values, traits and behaviours that have traditionally been taken as indicators of morality by a wide range of US Americans (for example, kindness, honesty, respect). We excluded from our analyses items that asked participants about their perceptions of special topics whose moral relevance either changed considerably over time (for example, men holding doors for women) or differed substantially across members of the population (for example, attending church). We also excluded items that asked participants about the morality of special subpopulations (for example, ‘Evangelicals’ or ‘the Wisconsin legislature’) rather than about all US Americans or about people in general. Further information, including search terms and all survey items included in study 1, can be found in the Supplementary Information. We also sampled our database for survey items administered to participants who lived outside the United States. Because there were fewer such surveys, we did not exclude surveys with non-representative samples, as we did with our US sample.

### Study 2a

All original data collection in this and subsequent studies followed all ethical regulations and was approved by the Institutional Review Board of Harvard University.

#### Participants

We recruited a nationally representative sample of US American adults using Prolific, an online sample provider. This sample was constructed to represent the US American adult population in terms of gender, race and age. Because we did not know the size of the effect we were studying, we sought to make our sample comparable in size to the samples in study 1 by recruiting 1,000 participants. Nine-hundred and ninety-nine people (507 female, 487 male, 5 other, *M*_age_ = 45.74 years, 73% white, 13% Black, 7% Asian, 4% Hispanic, 1% American Indian or Alaska Native, 1% other, 2% ‘more than one of the above’) were paid US$0.75 each for their participation.

#### Procedure

Study 2a was conducted in 2020. After providing informed consent, participants confirmed their Prolific ID, per the site’s usage policy. They then read the following instructions: “Thanks! In this study, we’ll ask you how kind, honest, nice, and good people were at various points in time. If you’re not sure, that’s okay, just give your best guess”. Participants then rated how “kind, honest, nice, and good” people are today, were 10 years ago and were 20 years ago, using seven-point Likert scales with endpoints labelled ‘not very’ and ‘very’. As a consistency check, participants were then asked to recall whether they had given higher, equal or lower ratings to people today compared to people 20 years ago. Participants then answered some open-ended exploratory questions that asked them to explain the thinking behind their answers. Participants then answered some demographic questions (Supplementary Table 6). Embedded among these demographic questions was an ‘attention check question’ that instructed participants to select the option ‘other’ and to type the word ‘sky’. Finally, participants were compensated and dismissed.

#### Exclusions

One hundred and eighty-one participants failed the attention check embedded in the demographics and were excluded from all analyses. Another 120 participants gave answers to the consistency check question that were inconsistent with their previous answers; they were also excluded. This left 698 participants in all analyses (372 female, 322 male, four other, *M*_age_ = 46.37, 74% white, 12% Black, 6% Asian, 4% Hispanic, 1% American Indian or Alaska Native, 2% more than one of the above). These exclusions do not meaningfully affect the results.

#### Analysis

To analyse the data, we fit a linear mixed effects model using the lme4 package in R^30^, extracted *P* values using the lmerTest package^31^ and calculated planned contrasts using the emmeans package^32^, using a Holm–Bonferroni correction for multiple comparisons. The outcome was participants’ ratings and the predictor was the year of those ratings (one factor with three levels: 2020, 2010 and 2000). The model included a fixed effect of the year of each rating and a random intercept for each participant. For this and all models, we checked model assumptions by plotting the outcome variable, residuals and fitted values. All tests we report are two-tailed.

### Study 2b

#### Participants

We powered study 2b to detect an effect of *d* = 0.30 or larger, reasoning that this would be sufficient to detect effects similar to the effect we detected in Study 2a. Two-hundred and thirty-six people responded to an advertisement for a study on Amazon Mechanical Turk. To participate, respondents had to pass a three-item test that required them to know that (1) children in kindergarten are 3 or 4 years old, (2) a US American ZIP code is a series of five digits and (3) eating turkey is not associated with Halloween. Thirty-six respondents answered at least one of these three questions incorrectly and were not allowed to participate. The remaining 200 respondents (81 female, 119 male, *M*_age_ = 35.81 years, 72% white, 12% Black, 9% Hispanic, 6% Asian, 3% more than one of the above) were allowed to participate in the study in exchange for US$0.75.

#### Procedure

After providing informed consent, participants followed study 2a’s procedure except they were asked about different years. Specifically, participants were first asked, “How kind, honest, nice, and good are people today?” and were then asked the same question for “two years ago”, “four years ago”, “six years ago”, “eight years ago” and “ten years ago”, in that order. All questions were answered using a seven-point Likert scale with endpoints labelled ‘not very’ and ‘very’. As a consistency check, participants then answered the following question: “When it comes to being kind, honest, nice, and good—are people more so today compared to ten years ago, less so today compared to ten years ago, or the same?” Participants were then asked to explain their answer in an open-ended question. Finally, participants were asked some demographic questions, as well as an attention check question that required them to select the option ‘other’ and to type the word ‘day’. Participants were compensated and dismissed.

#### Exclusions

Fifteen participants failed the attention check, and a further 37 participants failed the consistency check by giving an answer that was inconsistent with their scale ratings. The data from these participants were excluded from all analyses, leaving 148 participants (59 female, 89 male, *M*_age_ = 36.59 years, 75% white, 9% Black, 7% Hispanic, 5% Asian, 1% Hawaiian or Pacific Islander, 3% more the one of the above). These exclusions only meaningfully affect the results in one case, namely, that when all participants are included, the difference between 2020 and 2016 is not significant.

#### Analysis

We fit the same model we fit in study 2a except that in this case the factor in the model had six levels (2020, 2018, 2016, 2014, 2012 and 2010).

### Study 2c

#### Participants

We sought to recruit a sample of people who varied widely in terms of age. As such, we created a survey with a quota of 50 participants in each of the following age groups: 18–24, 25–29, 30–34, 35–39, 40–44, 45–49, 50–54, 55–59, 60–64 and 65–69 years. This sample size gave us sufficient power to detect the effects we had detected in studies 2a and 2b. Respondents selected their age group on accessing the study, and once the quota for a group was reached, further respondents from that group were not allowed to participate. Respondents younger than 18 or older than 69 were not allowed to participate.

Respondents responded to an advertisement for a study on Amazon Mechanical Turk. Respondents who accessed the survey before the quota for their age group was reached were asked to complete a three-item test of English proficiency and knowledge of US American culture. Specifically, they were required to demonstrate that they knew that (1) bell bottoms are not a type of footwear, (2) an RSVP is a required response to a wedding invitation and (3) a sign reading ‘out of order’ is best paired with an elevator. Three hundred and one respondents answered one or more of these questions incorrectly and were not allowed to participate. The remaining 484 respondents (225 female, 257 male, two other, *M*_age_ = 41.27 years, 72% white, 15% Black, 7% Asian, 4% Hispanic, 1% American Indian or Alaska Native, 2% more than one of the above) were allowed to participate in the study in exchange for US$0.75.

#### Procedure

Study 2c was conducted in 2020. Participants responded to an advertisement for a study on Amazon Mechanical Turk. After providing informed consent, participants reported how “kind, honest, nice and good” people are today. They then reported how “kind, honest, nice and good” people were when they (the participants) were about 20 years old, and at about the time they (the participants) were born. This was done by adjusting the wording of the subsequent questions on the basis of the participant’s age. For example, if the participant was between 30 and 34 years old, they were asked “How kind, honest, nice, and good were people about ten years ago?” and then “How kind, honest, nice, and good were people about 30 years ago?” If participants were under 25 years, they answered only the questions for today and when they were born. All questions were answered using a seven-point Likert scale with endpoints labelled ‘not very’ and ‘very’. As in previous studies, participants were then given a consistency check that required them to remember whether they had rated people today as more, equally or less moral compared to people in the year they were born. Participants then answered some further exploratory and demographic questions. Embedded among them was an attention check that required participants to select the option ‘other’ and type the word ‘apple’. Finally, participants were compensated and dismissed.

#### Exclusions

Twenty-eight participants failed the attention check and their data were excluded from all analyses. Seventy-three more participants reported an age at the end of the study that was inconsistent with the age group they selected at the beginning of the study and the data from these participants were also excluded from all analyses. An extra 64 participants failed the consistency check and data from these participants were also excluded from all analyses. The data from the remaining 347 participants (174 female, 172 male, one other, *M*_age_ = 42.57 years, 78% white, 9% Black, 7% Asian, 4% Hispanic, 2% ‘more than one of the above’) were included in all analyses. These exclusions do not meaningfully change the results.

#### Analysis

We fit the same model we fit in study 2b except that in this case the factor in the model had three levels (today, the year the participant turned 20, the year the participant was born).

### Study 3

#### Participants

Respondents responded to an advertisement for a study on Amazon Mechanical Turk. As in study 2c, we sought to recruit a sample of people who varied widely in terms of age and that was large enough to provide sufficient power to detect the effects we had detected in studies 2a and 2b. We created a survey with quota of 150 for each of three age groups: 20–34, 35–49 and 50–64. Anyone younger than 20 or older than 64 was not allowed to participate. Respondents were asked to complete the same test of English language and US American culture as in study 2c. Four hundred and forty-four respondents (202 female, 242 male, *M*_age_ = 40.42 years, 77% white, 9% Black, 7% Asian, 5% Hispanic, 1% ‘more than one of the above’) provided informed consent and became participants in the study in exchange for US$0.75.

#### Procedure

Study 3 was conducted in 2020. After providing informed consent, participants reported how “kind, honest, nice, and good” people are in the present (2020) and also “about 15 years ago” (about 2005) on seven-point Likert scales with endpoints labelled ‘not very’ and ‘very’ and then completed a consistency check that asked them to recall the answers they had just given. The difference between these two ratings was used as a measure of participants’ perception of moral decline between 2005 and 2020. Participants then answered the following questions using the same seven-point Likert scales: “How kind, honest, nice, and good are people who are currently between the ages of 35 and 95?”; “How kind, honest, nice, and good are people who are currently between the ages of 20 and 35?”; “Thinking again of people who are currently between the ages of 35 and 95, how kind, honest, nice, and good were they about 15 years ago?” and “About 15 years ago, how kind, honest, nice, and good were people who were then between the ages of 80 and 95?” Participants then answered some demographics questions, among which was embedded an ‘attention check question’ that instructed participants to select the option ‘other’ and to type the word ‘cloud’. Finally, participants were compensated and dismissed.

#### Exclusions

Forty-eight participants failed the attention check, and a further 15 participants reported an age at the end of the study that was inconsistent with the age group they reported at the beginning of the study. An extra 77 participants failed the consistency check. The data from all of these participants were excluded from all analyses, leaving 319 participants (154 female, 165 male, *M*_age_ = 41.02, 77% white, 8% Black, 8% Asian, 5% Hispanic, 1% more than one of the above). These exclusions do not meaningfully affect the results.

#### Calculating personal change and interpersonal replacement

**Extended Data Fig. 1:**
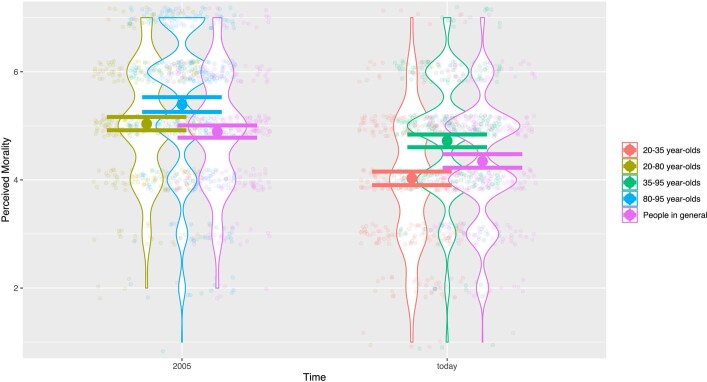
Descriptive Statistics in Study 3. Error bars represent 95% confidence intervals. Transparent points represent individual observations jittered for legibility.

We created a personal change score by subtracting ratings of 20–80-year olds about 15 years ago (in 2005) from ratings of 35–95-year olds in the present (2020). We created an interpersonal replacement score by subtracting ratings of 80–95-year olds about 15 years ago (in 2005) from ratings of 20–35-year olds in the present (2020). The descriptive statistics for people in general and each of the subgroups about which participants were asked are shown in Extended Data Fig. 1.

#### Analysis

Using a standard linear model, we entered participants’ personal change and cohort replacement scores as predictors, and the outcome was participants’ overall perception of moral decline between 2005 and 2020.

### Study 4

In study 4, we conducted keyword-term searches of the Roper Center for Public Opinion iPoll Database (using search terms shown in the Supplementary Information), and manually searched the databases of the General Social Survey, Pew Research Center, Gallup, the American National Election Studies, the World Values Survey, the European Social Survey and the European Values Survey to locate survey items that asked participants questions about their own and other people’s morality. As in study 1, questions were considered relevant to morality if they asked about values, attitudes, traits and behaviours that we thought would be considered relevant to kindness, honesty, niceness and goodness by a wide range of US Americans. We included US samples only if they were nationally representative, but also collected non-representative samples if they were collected outside the United States to maximize non-US representation. The latter were analysed separately. To be included, each survey had to be administered at least twice, and the most recent administration could not be earlier than 2010. Further information, including search terms and all survey items included in study 4, can be found in the Supplementary Information.

#### Analysis

We fit a linear model for each survey. The year of each survey was always entered as a predictor and the outcome was always the average perception of current morality. We used *R*^2^ values as a measure of effect size. We fit Bayesian models using the Rstanarm package in R^33^ and extracted the percentage of the 89% HDI that was contained in the ROPE, which was by default defined as ±0.1 standard deviations. We used the package’s default Markov Chain Monte Carlo and prior settings (*M* = 0, scale of 2.5).

### Study 5a

#### Participants

As in study 2c, we sought to recruit a sample of people who varied widely in terms of age and that was large enough to provide sufficient power to detect the effects we had detected in previous studies. We created a survey with a quota of 50 participants in each of three age groups: 20–34, 35–49 and 50–64 years. Anyone who was either younger than 20 years or older than 64 years was not allowed to participate.

One thousand and twenty-one people responded to an advertisement for a study on Amazon Mechanical Turk. They completed the same test of English language and US American culture as in study 2c. Five hundred and twenty-one respondents answered at least one of the questions incorrectly and were not allowed to participate. The remaining 500 respondents (204 female, 293 male, three other, *M*_age_ = 37.74 years, 65% white, 24% Black, 7% Asian, 2% Hispanic, 1% American Indian or Alaska Native, 1% more than one of the above) provided informed consent and became participants in the study in exchange for US$0.75.

#### Procedure

Study 5a was conducted in 2021. After providing informed consent, participants completed the same procedure as was used in study 2c, with two more questions. Specifically, participants rated how “kind, honest, nice, and good” people in general were 20 years before the participant was born and also 40 years before the participant was born. These years were adjusted on the basis of the age of the participant.

#### Exclusions

One hundred and seventy-nine participants failed the first attention check, and another 21 failed the second attention check. Another 15 participants reported an age at the end of the study that was inconsistent with the birth year they reported at the beginning. The data from all these participants were excluded from all analyses. The remaining 283 participants (139 female, 143 male, one other, *M*_age_ = 38.77 years, 78% white, 11% Black, 8% Asian, 2% Hispanic, 1% more than one of the above) were included in all analyses. These exclusions affect the results in a few cases. Specifically, when excluded participants are included, the overall perception of moral decline and personal change for people in general are not significant. All other effects remain significant.

#### Analysis

We fit the same model we fit in study 2c except that the factor in the model had five levels (2020, the year the participant turned 20, the year the participant was born, 20 years before the participant was born and 40 years before the participant was born).

### Study 5b

#### Participants

Because this study was a replication and extension of study 2c, we sought to collect a similar sample size to have the power to detect similar effects, and we used the same age quotas as in Study 2c. One thousand eighty-two people responded to an advertisement for a study on Amazon Mechanical Turk. Twenty-one of these opened the study but did not complete it. Five hundred and sixty people responded after the quota for their age group had been reached and were not allowed to participate in the study. Respondents who responded before the quota for their age group was reached completed the same three-item test of US American culture and English language used in study 2c. Twenty-three respondents answered one or more of these questions incorrectly and were not allowed to participate in the study. The remaining 499 respondents (225 female, 241 male, three other, *M*_age_ = 43.96 years, 78% white, 10% Asian, 5% Black, 4% Hispanic, 3% more than one of the above) were allowed to participate in the study in exchange for US$0.75.

#### Procedure

Study 5b was conducted in 2021. After providing informed consent, participants completed the same procedure used in study 2c. They further rated people’s morality 20 and 40 years before the year that they were born.

#### Exclusions

Forty-four participants failed the attention check and their data were excluded from all analyses. Seven more participants reported an age at the end of the study that was inconsistent with the age group they selected at the beginning of the study and their data were also excluded from all analyses. Sixty-one more participants failed the consistency check and their data were also excluded from all analyses. The data from the remaining 387 participants (206 female, 178 male, three other, *M*_age_ = 44.04 years, 79% white, 11% Asian, 4% Black, 3% Hispanic, 2% more than one of the above) were included in all analyses. These exclusions affect the results in one case: when excluded participants are included, participants perceived moral improvement from 40 years before birth to 20 years before birth. All other effects remain the same.

#### Analysis

We fit the same model we fit in study 2c except that in this case the factor in the model had five levels (the year 2020, the year the participant turned 20 years old, the year the participant was born, 20 years before the participant was born and 40 years before the participant was born).

### Reporting summary

Further information on research design is available in the Nature Portfolio Reporting Summary linked to this article.

## Online content

Any methods, additional references, Nature Portfolio reporting summaries, source data, extended data, supplementary information, acknowledgements, peer review information; details of author contributions and competing interests; and statements of data and code availability are available at 10.1038/s41586-023-06137-x.

## Supplementary information

Supplementary Information

Reporting Summary

## Data Availability

All materials and original data are available at https://osf.io/t83zy/ (10.17605/OSF.IO/T83ZY). The data analysed in studies 1 and 4 are the property of the polling organizations that produced them and cannot be posted. Instructions for accessing these data are also available at https://osf.io/t83zy/.

## Code availability

The code necessary to reproduce all analyses is available at https://osf.io/t83zy/, except for studies 1 and 4, which requires access to proprietary data.
