# Supplementary material for: The illusion of moral decline
Source: Nature. 2023 Jun 7;618(7966):782–9. doi: 10.1038/s41586-023-06137-x (PMC10284688; doi:10.1038/s41586-023-06137-x)
Supplement: Supplementary file 1 — Supplementary Information [file 41586_2023_6137_MOESM1_ESM.docx]

Supplemental Information

[Section 1: Additional Details of Studies Appearing in Main Text 2](#_Toc133250761)

[Search Terms Used in Studies 1 and 4 2](#_Toc133250762)

[A Note on the Calculation of the Grand Means in Study 1 2](#_Toc133250763)

[Table S1: Archival Survey Questions (US Sample), Study 1 4](#_Toc133250764)

[Table S2: Archival Survey Questions (non-US Sample), Study 1 27](#_Toc133250765)

[Table S3: Archival Survey Questions (US Sample) and Results, Study 4 35](#_Toc133250766)

[Table S4: Archival Survey Questions (non-US Sample) and Results, Study 4 63](#_Toc133250767)

[Additional Analyses in Study 5a 70](#_Toc133250768)

[Table S5: Regression Coefficients for Each of the Four Models Fit in Study 5a 71](#_Toc133250769)

[Table S6: Demographics Questions Used in Studies 2a-c, 3, and 5a-b 72](#_Toc133250770)

[Section 2: Additional Studies Not Appearing in Main Text 73](#_Toc133250771)

[Study S1: Pre-registered Replication of Study 2a 73](#_Toc133250772)

[Study S2: Replication of Study 2a with Participants from MTurk 74](#_Toc133250773)

[Study S3: Estimated Rates of Cooperation in Prisoner’s Dilemma Games (1956-2017) 75](#_Toc133250774)

[Section 3: Discussion of Related Literature 80](#_Toc133250775)

[Other Demonstrations of the Perception of Moral Decline 80](#_Toc133250776)

[Potential Causes of the Perception of Moral Decline 83](#_Toc133250777)

[Section 4: Mathematical Model of the BEAM Mechanism 85](#_Toc133250778)

[Section 5: References in Supplemental Information Section 90](#_Toc133250779)

Section 1: Additional Details of Studies Appearing in Main Text

Search Terms Used in Studies 1 and 4

We searched the following databases (hyperlinked to source): [General Social Survey](https://gss.norc.org/), [Pew Research Center](https://www.pewresearch.org/), [Gallup](https://www.gallup.com/home.aspx), the [American National Election Studies](https://electionstudies.org/data-center/), the [World Values Survey](https://www.worldvaluessurvey.org/wvs.jsp), the [European Social Survey](https://www.europeansocialsurvey.org/), and the [European Values Survey](https://europeanvaluesstudy.eu/). In addition, we queried the [Roper Center for Public Opinion](https://ropercenter.cornell.edu/ipoll/) using the following search terms: *moral/morality, kind/kindness, honest/honesty, nice/niceness, good/goodness, polite/politeness, rude/rudeness, ethics/ethical, lazy/laziness, hardworking, selfish/selfishness/selflessness, trust/trustworthy/untrustworthy, values, standards, safer place.*

In Study 1, to find surveys that specifically asked participants to compare people in the present to people at some point in the past, we also combined the above search terms with the following terms: *compared, generation, today, Years ago, worse/better/same, more/less/same, past.* We also searched for surveys that had the following tags in the Roper iPoll system: *morality, civility, crime rate, young people, values*. This search was conducted over several months in 2019 and 2020, before Roper iPoll transitioned to a new database and search interface, which may now return slightly different results.

A Note on the Calculation of the Grand Means in Study 1

There are two ways to compute the grand mean of the surveys included in Study 1, and each has a unique problem. One method is to weight the result of each survey by its sample size before averaging across surveys (the *weighted method*) and the other is to average the results across surveys without regard to the survey’s sample size (the *unweighted method*). The problem with the weighted method is that it can allow items with unusual wordings or unusual content to have an outsize influence on the grand mean simply because the survey containing those items happened to have had large N’s. The problem with the unweighted method is that it allows items from surveys with very small Ns (whose results are less trustworthy) to have the same influence on the grand mean as do surveys with very large Ns (whose results are more trustworthy).

We used the unweighted method because, in the present case, the first of these problems is significant and the second of these problems is not. Specifically, as Tables S1 and S2 show, the items included in Study 1 differed quite dramatically in terms of their wording and their content (e.g., “Would you say, compared to the past, people of different races and ethnicities are now treated much more equally, a little more equally, a little less equally, much less equally, or that there has been no real change?” vs. “Compared with 20 years ago, has the rate of violent crime in the United States increased or decreased?”). It seemed important that conclusions about the general perception of moral decline not be overly influenced by a few unusual items that happened to be worded in unusual ways or that happened to focus on particular topics. In contrast, although N did differ across surveys, all Ns were quite substantial, which is to say that even the surveys with the smallest N’s provided trustworthy results. When all results are trustworthy, then the unweighted method’s problem is not a problem, but its strengths are still strengths. For these reasons, we used the unweighted method to calculate the grand means in Study 1.

With all this said, it is worth noting that the weighted and unweighted methods produced the same general result. In both cases, the majority of participants perceived moral decline (61.44% using the weighted approach and 84.18% using the unweighted approach).

Table S1: Archival Survey Questions (US Sample), Study 1

| **#** | **Source** | **Question** | **Year** | **N** |
| --- | --- | --- | --- | --- |
| 1 | Pew | (As I read a list of some problems, please tell me if you think the country is making progress, losing ground, or if things are about the same as they have been.)...Low moral and ethical standards...Do you think the country is making progress, losing ground, or are things about the same as they have been when it comes to...low moral and ethical standards? | 2008 | 1489 |
| 2 | NBC News/Wall Street Journal | (For each of the decades I name, please tell me whether you feel the nation's morals and values were much higher in that decade than they are now, somewhat higher than now, somewhat lower than now, or much lower than they are now.)... The 1960s | 1996 | 2003 |
| 3 | NBC News/Wall Street Journal | (For each of the decades I name, please tell me whether you feel the nation's morals and values were much higher in that decade than they are now, somewhat higher than now, somewhat lower than now, or much lower than they are now.)... The 1970s | 1996 | 2003 |
| 4 | NBC News/Wall Street Journal | (For each of the decades I name, please tell me whether you feel the nation's morals and values were much higher in that decade than they are now, somewhat higher than now, somewhat lower than now, or much lower than they are now.)... The 1980s | 1996 | 2003 |
| 5 | Center for Survey Research, University of Virginia | (How strong would you say the US (United States) decline or improvement is in each of the following areas...strong decline, moderate decline, holding steady, moderate improvement, or strong improvement?)... American moral and ethical standards | 1996 | 2047 |
| 6 | NORC | (I'd like to ask you a few questions about civility in our society. By civility, I mean showing respect for the people you deal with. For example, respecting other people's opinions, being courteous, helping others, and showing good sportsmanship.)...In general, when most Americans debate issues facing the country do you think they are more civil today compared to 10 years ago, less civil, or about as civil as they were 10 years ago? | 2011 | 1006 |
| 7 | Gallup | (I'm going to read you several more statements and ask you whether, in general, you agree or disagree.)... Moral and ethical standards are declining so much in America these days that something drastic must be done. | 1964 | 1564 |
| 8 | Pew | (Now I'd like to ask some questions about some of the problems we face in this country today. For each problem I mention, please tell me how you think affecting this country today, and how much this problem affects you personally.)...Do you think the problem of...low moral and ethical standards is about the same as it has been, that the country is making progress in this area, or that the country is losing ground? | 1996 | 1204 |
| 9 | NBC News/Wall Street Journal | (Over the past decade of 2000 to 2009, do you feel that America has gained ground, has stayed about the same, or has lost ground in each of the following areas?)...Moral values | 2009 | 1008 |
| 10 | NBC News/Wall Street Journal | (Over the past decade of 2000 to 2009, do you feel that America has gained ground, has stayed about the same, or has lost ground in each of the following areas?)...Treating one another with respect | 2009 | 1008 |
| 11 | Center for Survey Research, University of Virginia | (Please consider the following statements and tell me whether you completely agree, mostly agree, mostly disagree, or completely disagree with each statement.)... In general, Americans lived more moral and ethical lives 50 years ago. | 1996 | 2047 |
| 12 | NBC News | (Thinking about where America stands today as compared to four years ago, for each of the following things, please tell me whether you think that things are better off, about the same, or worse off than they were four years ago.)...Moral values and standards | 2005 | 1007 |
| 13 | NBC/Wall Street Journal | Compared to ten years ago, would you say that Americans now are more polite to each other or less polite to each other? | 1999 | 1006 |
| 14 | NORC | Compared with 10 years ago, would you say that when it comes to...the willingness of people to help each other, things in this country have generally gotten better, gotten worse, or stayed about the same? (If Better/Worse, ask:) Is that a lot better/worse or a little better/worse? | 2011 | 1087 |
| 15 | NORC | Compared with 10 years ago, would you say that when it comes to...the willingness of people to help each other, things in this country have generally gotten better, gotten worse, or stayed about the same? Is that a lot better/worse or a little better/worse? | 2013 | 1008 |
| 16 | Harris Survey | Compared with ten years ago, would you say morality in the United States is lower today, higher, or not changed much? | 1971 | 1600 |
| 17 | CBS News/New York Times | Considering just the moral climate of the country today, do you feel things in this country are generally going in the right direction or do you feel things have pretty seriously gotten off on the wrong track? | 2004 | 885 |
| 18 | Pew | Do you think that Americans used to treat each other with more respect and courtesy in the past, or is this just nostalgia for a past that never existed? | 2002 | 2013 |
| 19 | Parents Magazine Poll | Do you think that over the last few decades our society has become less honest and ethical in its behavior, more honest and ethical, or has there been no change in the extent to which people behave honestly and ethically? | 1989 | 1000 |
| 20 | Gallup | Generally speaking, do you think moral values have become stronger in the U.S. (United States) in the last twenty-five years, do you think they have become weaker or do you think they have stayed about the same? | 1996 | 1001 |
| 21 | Pew | Here is a list of things that may or may not be problems in our country. As I read each one, please tell me if you think it is a very big problem, a moderately big problem, a small problem, or not a problem at all. Moral decline. | 2006 | 739 |
| 22 | Time/Yankelovich | Now here are some statements which represent some traditional American values. Will you tell me for each one whether you strongly believe in this statement, partially believe it or don't believe it. The state of morality in this country is bad and getting worse. | 1976 | 951 |
| 23 | Center for Survey Research and Analysis, University of Connecticut | Overall, would you say that the moral values in American society are improving, deteriorating, or aren't they changing all that much? | 1997 | 1026 |
| 24 | Gallup | Right now, do you think the state of moral values in this country as a whole is getting better or getting worse? | 2019 | 1009 |
| 25 | Gallup | Right now, do you think the state of moral values in this country as a whole is getting better or getting worse? | 2018 | 1024 |
| 26 | Gallup | Right now, do you think the state of moral values in this country as a whole is getting better or getting worse? | 2017 | 1011 |
| 27 | Gallup | Right now, do you think the state of moral values in this country as a whole is getting better or getting worse? | 2016 | 1025 |
| 28 | Gallup | Right now, do you think the state of moral values in this country as a whole is getting better or getting worse? | 2015 | 1024 |
| 29 | Gallup | Right now, do you think the state of moral values in this country as a whole is getting better or getting worse? | 2014 | 1028 |
| 30 | Gallup | Right now, do you think the state of moral values in this country as a whole is getting better or getting worse? | 2013 | 1535 |
| 31 | Gallup | Right now, do you think the state of moral values in this country as a whole is getting better or getting worse? | 2012 | 1024 |
| 32 | Gallup | Right now, do you think the state of moral values in this country as a whole is getting better or getting worse? | 2011 | 1018 |
| 33 | Gallup | Right now, do you think the state of moral values in this country as a whole is getting better or getting worse? | 2010 | 1029 |
| 34 | Gallup | Right now, do you think the state of moral values in this country as a whole is getting better or getting worse? | 2009 | 1015 |
| 35 | Gallup | Right now, do you think the state of moral values in this country as a whole is getting better or getting worse? | 2008 | 1017 |
| 36 | Gallup | Right now, do you think the state of moral values in this country as a whole is getting better or getting worse? | 2007 | 1003 |
| 37 | Gallup | Right now, do you think the state of moral values in this country as a whole is getting better or getting worse? | 2006 | 1002 |
| 38 | Gallup | Right now, do you think the state of moral values in this country as a whole is getting better or getting worse? | 2005 | 1005 |
| 39 | Gallup/CNN/USA Today | Right now, do you think the state of moral values in this country as a whole is getting better or getting worse? | 2004 | 1015 |
| 40 | Gallup | Right now, do you think the state of moral values in this country as a whole is getting better or getting worse? | 2004 | 1000 |
| 41 | Gallup | Right now, do you think the state of moral values in this country as a whole is getting better or getting worse? | 2003 | 1005 |
| 42 | Gallup | Right now, do you think the state of moral values in this country as a whole is getting better or getting worse? | 2002 | 1012 |
| 43 | Opinion Research Corporation | Some people say there has been a general letdown in ethical and moral standards among the American people as a whole in recent years. Others say ethical and moral standards are as good as ever. Which do you agree with? | 1964 | 1021 |
| 44 | Time/Yankelovich | The following are a number of criticisms that have been made in recent years about American society. Will you tell me for each one how you personally feel? The state of morals in this country are pretty bad and getting worse. | 1974 | 1238 |
| 45 | NBC News/Wall Street Journal | Thinking back to when you were growing up, would you say that the social and moral values in the United States were the same as today, higher than today, or lower than today? | 1999 | 2011 |
| 46 | Market Strategies | Thinking specifically about the state of the country's morals and values, do you feel things are generally going in the right direction, or do you feel things have pretty seriously gotten off on the wrong track? | 1999 | 800 |
| 47 | NBC News/Wall Street Journal | Which of the following statements comes closest to expressing how you feel about the state of morals in this country at the present time... they are pretty bad and getting worse, they are pretty bad but getting better, they are pretty good but getting worse, or they are pretty good and getting better? | 1996 | 2003 |
| 48 | NORC | Which of the statements on this card comes closest to expressing how you feel about the state of morals in this country at the present time? They are pretty bad and getting worse, they are pretty bad but getting better, they are pretty good but getting worse, they are pretty good and getting better | 1964 | 1975 |
| 49 | NBC News/Wall Street Journal | Which one of the following statements comes closest to expressing how you feel about the state of morals in this country at the present time?...They are pretty bad and getting worse, they are pretty bad but getting better, they are pretty good but getting worse, they are pretty good and getting better | 2004 | 1003 |
| 50 | YouGov - PTN | Thinking about the nation as a whole, do you think crimes motivated by hatred (racist, anti-religious, homophobic and anti-ethnic) over the past 12 months are higher or lower compared to ten years ago? | 2019 | 1500 |
| 51 | Economist - PTN | Thinking about the nation as a whole, do you think the number of violent crime incidents (homicide, sexual assault, robbery, serious assault) over the past 12 months is higher or lower compared to ten years ago? | 2017 | 2692 |
| 52 | Economist - PTN | In the last year would you say crime in the country has increased, decreased, or stayed about the same? | 2017 | 2692 |
| 53 | Economist - PTN | Thinking about the nation as a whole, do you think the number of violent crime incidents (homicide, sexual assault, robbery, serious assault) over the past 12 months is higher or lower compared to ten years ago? | 2017 | 2692 |
| 54 | YouGov - PTN | Compared with 20 years ago, has the rate of violent crime in the United States increased or decreased? | 2014 | 1000 |
| 55 | Kaiser Family Foundation | Compared with 20 years ago, has the rate of violent crime in the United States increased or decreased? | 1996 | 1514 |
| 56 | Gallup - PTN | Has the crime rate in the US increased, decreased, or stayed the same in the past 10 years? | 1989 | 1235 |
| 57 | Barna Research Group - PTN | Percentage of Americans who believe the values and morals of America are declining. | 2013 | 2083 |
| 58 | NBC News - PTN | Now I would like to read you several more statements about some of the social issues facing America. For each issue, please tell me whether you agree strongly, agree somewhat, disagree somewhat, or disagree strongly with that statement: traditional moral values have grown weaker and need to be strengthened | 1993 | 1502 |
| 59 | Marist | Do you think Americans overall are more respectful, less respectful, or about as respectful of each other as they were a few years ago? | 2019 | 1084 |
| 60 | NPR | From what you have read or heard, do you think, compared to 25 years ago, the per capita gun murder rate in the U.S. is higher, lower, or about the same? | 2019 | 880 |
| 61 | Economist | Compared with 20 years ago, has the number of gun crimes in America gone up, gone down or stayed the same? | 2013 | 1000 |
| 62 | Pew | (And compared to the past, have things gotten better, worse or stayed the same?)...Being friendly and helpful toward their neighbors | 2002 | 2013 |
| 63 | Pew | (And compared to the past, have things gotten better, worse or stayed the same?)...Being kind and considerate toward people with physical handicaps | 2002 | 2013 |
| 64 | Pew | (And compared to the past, have things gotten better, worse or stayed the same?)...The problem of littering | 2002 | 2013 |
| 65 | Pew | (And compared to the past, have things gotten better, worse or stayed the same?)...Treating African Americans with respect and courtesy | 2002 | 2013 |
| 66 | Pew | (And compared to the past, have things gotten better, worse or stayed the same?)...Treating gay people with respect and courtesy | 2002 | 2013 |
| 67 | Pew | (And compared to the past, have things gotten better, worse or stayed the same?)...Treating Hispanics with respect and courtesy | 2002 | 2013 |
| 68 | Pew | (And compared to the past, have things gotten better, worse or stayed the same?)...Treating the elderly with respect and courtesy | 2002 | 2013 |
| 69 | Gallup | (Does the following word apply more to young people in their teens and 20s today or young people in that same age group 20 years ago?)... Materialistic | 1989 | 1249 |
| 70 | Gallup | (Does the following word apply more to young people in their teens and 20s today or young people in that same age group 20 years ago?)... Patriotic | 1989 | 1249 |
| 71 | Gallup | (Does the following word apply more to young people in their teens and 20s today or young people in that same age group 20 years ago?)... Reckless | 1989 | 1249 |
| 72 | Gallup | (Does the following word apply more to young people in their teens and 20s today or young people in that same age group 20 years ago?)... Selfish | 1989 | 1249 |
| 73 | CBS News | Compared to 10 years ago, do you think more people today are willing to take responsibility when they have done something wrong, fewer people today are willing to take responsibility when they have done something wrong, or hasn't this changed much in the past 10 years? | 1994 | 871 |
| 74 | Associated Press/Ipsos | Compared to 20 or 30 years ago, do you think people are more rude, less rude, or about the same? | 2005 | 1001 |
| 75 | NORC | Compared to 20 or 30 years ago, do you think people are more rude, less rude, or about the same? | 2016 | 1004 |
| 76 | Gallup | Compared to ten years ago, are people more honest, less honest, or about the same today? | 1987 | 1005 |
| 77 | Roper/US News & World Report | Compared to ten years ago, are people more honest, less honest, or about the same today? | 1987 | 1005 |
| 78 | LA Times | Do you believe that life today is getting better or worse in terms of morals? | 1985 | 2308 |
| 79 | Gallup | Do you believe that life today is getting better or worse in terms of: morals? | 1968 | 1536 |
| 80 | Washington Post/Harvard/Kaiser | Do you think people in general lead as good lives honest and moral as they used to? | 1998 | 1018 |
| 81 | Roper/US News & World Report | Do you think people in general today are more honest and moral than they were in the 1950's, or less honest and moral than then, or about the same as they were in the 1950's? | 1985 | 1003 |
| 82 | Pew | Do you think people in general today lead as good lives - honest and moral - as they used to? | 2002 | 2002 |
| 83 | Ben Gaffin and Associates | Do you think people in general today lead as good lives--honest and moral--as they used to? | 1952 | 2987 |
| 84 | Gallup | Do you think people in general today lead as good lives--honest and moral--as they used to? | 1965 | 2783 |
| 85 | Gallup | Do you think people in general today lead as good lives--honest and moral--as they used to? | 1976 | 1538 |
| 86 | Pew | Do you think people in general today lead as good lives--honest and moral--as they used to? | 2005 | 1505 |
| 87 | Gallup | Do you think that people today are more willing or less willing to help each other than they used to be, say ten years ago? | 1982 | 1729 |
| 88 | Louis Harris and Associates | Do you think that people's motivation to work today is stronger or not as strong as it was ten years ago? | 1980 | 1201 |
| 89 | Gallup | Do you think the human race is getting better or worse from the standpoint of moral conduct? | 1949 | 1500 |
| 90 | NBC News/Wall Street Journal | For each of the decades I name, please tell me whether you feel the nation's morals and values were much higher in that decade than they are now, somewhat higher than now, somewhat lower than now, or much lower than they are now.... The 1950s | 1996 | 2003 |
| 91 | Barna Research Group | How have people's attitudes, lifestyles and behaviors changed in the last 10 years: moral values? | 1993 | 687 |
| 92 | NBC | In general, do you think people today are a lot more honest, somewhat more honest, somewhat less honest, a lot less honest or about the same as 20 years ago? | 1996 | 504 |
| 93 | NBC News | In general, do you think people today are a lot more honest, somewhat more honest, somewhat less honest, a lot less honest or about the same as 20 years ago? | 1995 | 1009 |
| 94 | NBC News | In general, do you think people today are a lot more honest, somewhat more honest, somewhat less honest, a lot less honest or about the same as 20 years ago? | 1996 | 504 |
| 95 | Barna Report | When it comes to moral values, do you think things have gotten better, gotten worse, or stayed about the same compared to 10 years ago? | 1993 | 1205 |
| 96 | Barna Report | When it comes to people's selfishness, do you think things have gotten better, gotten worse, or stayed about the same compared to 10 years ago? | 1993 | 1205 |
| 97 | American Enterprise Institute | Would you say that people are more willing, less willing, or about as willing to help their neighbors as they were twenty-five years ago? | 1981 | 1500 |
| 98 | World Public Opinion - PTN | Thinking about the course of your lifetime, would you say, compared to the past, people of different races and ethnicities are now treated much more equally, a little more equally, a little less equally, much less equally, or that there has been no real change? | 2008 | 1819 |
| 99 | CBS News - PTN | In the last eight years, do you think crime has increased, decreased, or stayed about the same? | 1989 | 1533 |
| 100 | Associated Press | Compared to 20 or 30 years ago, do you think people are more rude, less rude, or about the same? | 2016 | 1004 |
| 101 | Associated Press | Compared to 20 or 30 years ago, do you think people are more rude, less rude, or about the same? | 2005 | NA |
| 102 | Pew | (Here is a list of things that may be problems in our country. As I read each one, please tell me if you think it is a very big problem, a moderately big problem, a small problem or not a problem at all.)...Moral decline | 2002 | 1501 |
| 103 | LA Times | Generally speaking, would you say the nation is undergoing a period of moral improvement, or a period of moral decline? | 1989 | 3583 |
| 104 | Roper | Here is a list of possible causes of some of our problems in this country. (Card shown respondent) Would you call off the ones you think are the major causes of our problems today? (Lack of good leadership, Permissiveness in the courts, Permissiveness of parents, Selfishness - people not thinking of others, Wrongdoing in government, Radical attempts to force change, Growing conservatism, Too much emphasis on money and materialism, Too much technology, A letdown in moral values, Too much commitment to other nations in the world, Too little interest in other nations in the world). | 1982 | 2000 |
| 105 | LA Times | What do you think is the single most urgent problem facing this country today: crime, or education, or the environment, or foreign trade, or government spending, or inflation, or the moral decline of society, or unemployment, or the fear of war, or what? I could repeat those, if you wish. Is there another one of those problems you consider almost as important? | 1989 | 2095 |
| 106 | Newsweek - PTN | Do you think the United States is in a moral and spiritual decline? | 1994 | 600 |
| 107 | Knight Ridder - PTN | Do you think the United States is in a moral and spiritual decline? | 1992 | 1387 |
| 108 | Marist College | In general, do you believe moral values in this country are headed in the right direction or the wrong direction? | 2009 | 2243 |
| 109 | Public Religion Research Institute | Now, as I read some statements on a few different topics, please tell me if you completely agree, mostly agree, mostly disagree or completely disagree with each one....The main cause of America's problems is moral decay. | 2012 | 3003 |
| 110 | TIPP/Investor's Business Daily/Christian Science Monitor | Okay, and generally speaking, how satisfied are you with the direction that the country is going in at this time in terms of morals and ethics? Would you say you are...very satisfied, somewhat satisfied, not very satisfied, or not at all satisfied? | 2004 | 1008 |
| 111 | TIPP/Investor's Business Daily/Christian Science Monitor | Okay, and generally speaking, how satisfied are you with the direction that the country is going in at this time in terms of morals and ethics? Would you say you are...very satisfied, somewhat satisfied, not very satisfied, or not at all satisfied? | 2019 | 903 |
| 112 | TIPP/Investor's Business Daily/Christian Science Monitor | Okay, and generally speaking, how satisfied are you with the direction that the country is going in at this time in terms of morals and ethics? Would you say you are...very satisfied, somewhat satisfied, not very satisfied, or not at all satisfied? | 2019 | 907 |
| 113 | TIPP/Investor's Business Daily/Christian Science Monitor | Okay, and generally speaking, how satisfied are you with the direction that the country is going in at this time in terms of morals and ethics? Would you say you are...very satisfied, somewhat satisfied, not very satisfied, or not at all satisfied? | 2019 | 903 |
| 114 | TIPP/Investor's Business Daily/Christian Science Monitor | Okay, and generally speaking, how satisfied are you with the direction that the country is going in at this time in terms of morals and ethics? Would you say you are...very satisfied, somewhat satisfied, not very satisfied, or not at all satisfied? | 2018 | 823 |
| 115 | TIPP/Investor's Business Daily/Christian Science Monitor | Okay, and generally speaking, how satisfied are you with the direction that the country is going in at this time in terms of morals and ethics? Would you say you are...very satisfied, somewhat satisfied, not very satisfied, or not at all satisfied? | 2018 | 900 |
| 116 | TIPP/Investor's Business Daily/Christian Science Monitor | Okay, and generally speaking, how satisfied are you with the direction that the country is going in at this time in terms of morals and ethics? Would you say you are...very satisfied, somewhat satisfied, not very satisfied, or not at all satisfied? | 2018 | 905 |
| 117 | TIPP/Investor's Business Daily/Christian Science Monitor | Okay, and generally speaking, how satisfied are you with the direction that the country is going in at this time in terms of morals and ethics? Would you say you are...very satisfied, somewhat satisfied, not very satisfied, or not at all satisfied? | 2018 | 929 |
| 118 | TIPP/Investor's Business Daily/Christian Science Monitor | Okay, and generally speaking, how satisfied are you with the direction that the country is going in at this time in terms of morals and ethics? Would you say you are...very satisfied, somewhat satisfied, not very satisfied, or not at all satisfied? | 2018 | 900 |
| 119 | TIPP/Investor's Business Daily/Christian Science Monitor | Okay, and generally speaking, how satisfied are you with the direction that the country is going in at this time in terms of morals and ethics? Would you say you are...very satisfied, somewhat satisfied, not very satisfied, or not at all satisfied? | 2018 | 905 |
| 120 | TIPP/Investor's Business Daily/Christian Science Monitor | Okay, and generally speaking, how satisfied are you with the direction that the country is going in at this time in terms of morals and ethics? Would you say you are...very satisfied, somewhat satisfied, not very satisfied, or not at all satisfied? | 2018 | 900 |
| 121 | TIPP/Investor's Business Daily/Christian Science Monitor | Okay, and generally speaking, how satisfied are you with the direction that the country is going in at this time in terms of morals and ethics? Would you say you are...very satisfied, somewhat satisfied, not very satisfied, or not at all satisfied? | 2018 | 902 |
| 122 | TIPP/Investor's Business Daily/Christian Science Monitor | Okay, and generally speaking, how satisfied are you with the direction that the country is going in at this time in terms of morals and ethics? Would you say you are...very satisfied, somewhat satisfied, not very satisfied, or not at all satisfied? | 2018 | 901 |
| 123 | TIPP/Investor's Business Daily/Christian Science Monitor | Okay, and generally speaking, how satisfied are you with the direction that the country is going in at this time in terms of morals and ethics? Would you say you are...very satisfied, somewhat satisfied, not very satisfied, or not at all satisfied? | 2018 | 900 |
| 124 | TIPP/Investor's Business Daily/Christian Science Monitor | Okay, and generally speaking, how satisfied are you with the direction that the country is going in at this time in terms of morals and ethics? Would you say you are...very satisfied, somewhat satisfied, not very satisfied, or not at all satisfied? | 2018 | 901 |
| 125 | TIPP/Investor's Business Daily/Christian Science Monitor | Okay, and generally speaking, how satisfied are you with the direction that the country is going in at this time in terms of morals and ethics? Would you say you are...very satisfied, somewhat satisfied, not very satisfied, or not at all satisfied? | 2017 | 901 |
| 126 | TIPP/Investor's Business Daily/Christian Science Monitor | Okay, and generally speaking, how satisfied are you with the direction that the country is going in at this time in terms of morals and ethics? Would you say you are...very satisfied, somewhat satisfied, not very satisfied, or not at all satisfied? | 2017 | 905 |
| 127 | TIPP/Investor's Business Daily/Christian Science Monitor | Okay, and generally speaking, how satisfied are you with the direction that the country is going in at this time in terms of morals and ethics? Would you say you are...very satisfied, somewhat satisfied, not very satisfied, or not at all satisfied? | 2017 | 904 |
| 128 | TIPP/Investor's Business Daily/Christian Science Monitor | Okay, and generally speaking, how satisfied are you with the direction that the country is going in at this time in terms of morals and ethics? Would you say you are...very satisfied, somewhat satisfied, not very satisfied, or not at all satisfied? | 2017 | 903 |
| 129 | TIPP/Investor's Business Daily/Christian Science Monitor | Okay, and generally speaking, how satisfied are you with the direction that the country is going in at this time in terms of morals and ethics? Would you say you are...very satisfied, somewhat satisfied, not very satisfied, or not at all satisfied? | 2017 | 901 |
| 130 | TIPP/Investor's Business Daily/Christian Science Monitor | Okay, and generally speaking, how satisfied are you with the direction that the country is going in at this time in terms of morals and ethics? Would you say you are...very satisfied, somewhat satisfied, not very satisfied, or not at all satisfied? | 2017 | 904 |
| 131 | TIPP/Investor's Business Daily/Christian Science Monitor | Okay, and generally speaking, how satisfied are you with the direction that the country is going in at this time in terms of morals and ethics? Would you say you are...very satisfied, somewhat satisfied, not very satisfied, or not at all satisfied? | 2017 | 904 |
| 132 | TIPP/Investor's Business Daily/Christian Science Monitor | In general, how satisfied are you with the direction that the country is going in at this time in terms of morals and ethics?...Very satisfied, somewhat satisfied, not very satisfied, not at all satisfied | 2017 | 909 |
| 133 | TIPP/Investor's Business Daily/Christian Science Monitor | How satisfied are you with the direction that the country is going in at this time in terms of morals and ethics?...Very satisfied, somewhat satisfied, not very satisfied, not at all satisfied | 2017 | 885 |
| 134 | TIPP/Investor's Business Daily/Christian Science Monitor | In general, how satisfied are you with the direction that the country is going in at this time in terms of morals and ethics?...Very satisfied, somewhat satisfied, not very satisfied, not at all satisfied | 2016 | 934 |
| 135 | TIPP/Investor's Business Daily/Christian Science Monitor | How satisfied are you with the direction that the country is going in at this time in terms of morals and ethics?...Very satisfied, somewhat satisfied, not very satisfied, not at all satisfied | 2016 | 921 |
| 136 | TIPP/Investor's Business Daily/Christian Science Monitor | How satisfied are you with the direction that the country is going in at this time in terms of morals and ethics?...Very satisfied, somewhat satisfied, not very satisfied, not at all satisfied | 2016 | 908 |
| 137 | TIPP/Investor's Business Daily/Christian Science Monitor | How satisfied are you with the direction that the country is going in at this time in terms of morals and ethics?...Very satisfied, somewhat satisfied, not very satisfied, not at all satisfied | 2016 | 902 |
| 138 | TIPP/Investor's Business Daily/Christian Science Monitor | How satisfied are you with the direction that the country is going in at this time in terms of morals and ethics?...Very satisfied, somewhat satisfied, not very satisfied, not at all satisfied | 2016 | 914 |
| 139 | TIPP/Investor's Business Daily/Christian Science Monitor | Okay, and generally speaking, how satisfied are you with the direction that the country is going in at this time in terms of morals and ethics? Would you say you are...very satisfied, somewhat satisfied, not very satisfied, or not at all satisfied? | 2004 | 1008 |
| 140 | TIPP/Investor's Business Daily/Christian Science Monitor | Okay, and generally speaking, how satisfied are you with the direction that the country is going in at this time in terms of morals and ethics? Would you say you are...very satisfied, somewhat satisfied, not very satisfied, or not at all satisfied? | 2004 | 938 |
| 141 | TIPP/Investor's Business Daily/Christian Science Monitor | Okay, and generally speaking, how satisfied are you with the direction that the country is going in at this time in terms of morals and ethics? Would you say you are...very satisfied, somewhat satisfied, not very satisfied, or not at all satisfied? | 2004 | 1003 |
| 142 | TIPP/Investor's Business Daily/Christian Science Monitor | Okay, and generally speaking, how satisfied are you with the direction that the country is going in at this time in terms of morals and ethics? Would you say you are...very satisfied, somewhat satisfied, not very satisfied, or not at all satisfied? | 2004 | 981 |
| 143 | TIPP/Investor's Business Daily/Christian Science Monitor | Okay, and generally speaking, how satisfied are you with the direction that the country is going in at this time in terms of morals and ethics? Would you say you are...very satisfied, somewhat satisfied, not very satisfied, or not at all satisfied? | 2004 | 1003 |
| 144 | TIPP/Investor's Business Daily/Christian Science Monitor | Okay, and generally speaking, how satisfied are you with the direction that the country is going in at this time in terms of morals and ethics? Would you say you are...very satisfied, somewhat satisfied, not very satisfied, or not at all satisfied? | 2004 | 958 |
| 145 | TIPP/Investor's Business Daily/Christian Science Monitor | Okay, and generally speaking, how satisfied are you with the direction that the country is going in at this time in terms of morals and ethics? Would you say you are...very satisfied, somewhat satisfied, not very satisfied, or not at all satisfied? | 2004 | 920 |
| 146 | TIPP/Investor's Business Daily/Christian Science Monitor | Okay, and generally speaking, how satisfied are you with the direction that the country is going in at this time in terms of morals and ethics? Would you say you are...very satisfied, somewhat satisfied, not very satisfied, or not at all satisfied? | 2003 | 905 |
| 147 | TIPP/Investor's Business Daily/Christian Science Monitor | Okay, and generally speaking, how satisfied are you with the direction that the country is going in at this time in terms of morals and ethics? Would you say you are...very satisfied, somewhat satisfied, not very satisfied, or not at all satisfied? | 2003 | 903 |
| 148 | TIPP/Investor's Business Daily/Christian Science Monitor | Okay, and generally speaking, how satisfied are you with the direction that the country is going in at this time in terms of morals and ethics? Would you say you are...very satisfied, somewhat satisfied, not very satisfied, or not at all satisfied? | 2003 | 901 |
| 149 | TIPP/Investor's Business Daily/Christian Science Monitor | Okay, and generally speaking, how satisfied are you with the direction that the country is going in at this time in terms of morals and ethics? Would you say you are...very satisfied, somewhat satisfied, not very satisfied, or not at all satisfied? | 2003 | 901 |
| 150 | TIPP/Investor's Business Daily/Christian Science Monitor | Okay, and generally speaking, how satisfied are you with the direction that the country is going in at this time in terms of morals and ethics? Would you say you are...very satisfied, somewhat satisfied, not very satisfied, or not at all satisfied? | 2003 | 901 |
| 151 | TIPP/Investor's Business Daily/Christian Science Monitor | Okay, and generally speaking, how satisfied are you with the direction that the country is going in at this time in terms of morals and ethics? Would you say you are...very satisfied, somewhat satisfied, not very satisfied, or not at all satisfied? | 2003 | 919 |
| 152 | TIPP/Investor's Business Daily/Christian Science Monitor | Okay, and generally speaking, how satisfied are you with the direction that the country is going in at this time in terms of morals and ethics? Would you say you are...very satisfied, somewhat satisfied, not very satisfied, or not at all satisfied? | 2003 | 906 |
| 153 | TIPP/Investor's Business Daily/Christian Science Monitor | Okay, and generally speaking, how satisfied are you with the direction that the country is going in at this time in terms of morals and ethics? Would you say you are...very satisfied, somewhat satisfied, not very satisfied, or not at all satisfied? | 2003 | 928 |
| 154 | TIPP/Investor's Business Daily/Christian Science Monitor | Okay, and generally speaking, how satisfied are you with the direction that the country is going in at this time in terms of morals and ethics? Would you say you are...very satisfied, somewhat satisfied, not very satisfied, or not at all satisfied? | 2003 | 900 |
| 155 | TIPP/Investor's Business Daily/Christian Science Monitor | Okay, and generally speaking, how satisfied are you with the direction that the country is going in at this time in terms of morals and ethics? Would you say you are...very satisfied, somewhat satisfied, not very satisfied, or not at all satisfied? | 2003 | 902 |
| 156 | TIPP/Investor's Business Daily/Christian Science Monitor | Okay, and generally speaking, how satisfied are you with the direction that the country is going in at this time in terms of morals and ethics? Would you say you are...very satisfied, somewhat satisfied, not very satisfied, or not at all satisfied? | 2002 | 900 |
| 157 | TIPP/Investor's Business Daily/Christian Science Monitor | Okay, and generally speaking, how satisfied are you with the direction that the country is going in at this time in terms of morals and ethics? Would you say you are...very satisfied, somewhat satisfied, not very satisfied, or not at all satisfied? | 2002 | 900 |
| 158 | TIPP/Investor's Business Daily/Christian Science Monitor | Okay, and generally speaking, how satisfied are you with the direction that the country is going in at this time in terms of morals and ethics? Would you say you are...very satisfied, somewhat satisfied, not very satisfied, or not at all satisfied? | 2002 | 912 |
| 159 | TIPP/Investor's Business Daily/Christian Science Monitor | Okay, and generally speaking, how satisfied are you with the direction that the country is going in at this time in terms of morals and ethics? Would you say you are...very satisfied, somewhat satisfied, not very satisfied, or not at all satisfied? | 2002 | 914 |
| 160 | TIPP/Investor's Business Daily/Christian Science Monitor | Okay, and generally speaking, how satisfied are you with the direction that the country is going in at this time in terms of morals and ethics? Would you say you are...very satisfied, somewhat satisfied, not very satisfied, or not at all satisfied? | 2002 | 903 |
| 161 | TIPP/Investor's Business Daily/Christian Science Monitor | Okay, and generally speaking, how satisfied are you with the direction that the country is going in at this time in terms of morals and ethics? Would you say you are...very satisfied, somewhat satisfied, not very satisfied, or not at all satisfied? | 2002 | 924 |
| 162 | TIPP/Investor's Business Daily/Christian Science Monitor | Okay, and generally speaking, how satisfied are you with the direction that the country is going in at this time in terms of morals and ethics? Would you say you are...very satisfied, somewhat satisfied, not very satisfied, or not at all satisfied? | 2002 | 900 |
| 163 | TIPP/Investor's Business Daily/Christian Science Monitor | Okay, and generally speaking, how satisfied are you with the direction that the country is going in at this time in terms of morals and ethics? Would you say you are...very satisfied, somewhat satisfied, not very satisfied, or not at all satisfied? | 2002 | 906 |
| 164 | TIPP/Investor's Business Daily/Christian Science Monitor | Okay, and generally speaking, how satisfied are you with the direction that the country is going in at this time in terms of morals and ethics? Would you say you are...very satisfied, somewhat satisfied, not very satisfied, or not at all satisfied? | 2002 | 900 |
| 165 | TIPP/Investor's Business Daily/Christian Science Monitor | Okay, and generally speaking, how satisfied are you with the direction that the country is going in at this time in terms of morals and ethics? Would you say you are...very satisfied, somewhat satisfied, not very satisfied, or not at all satisfied? | 2002 | 921 |
| 166 | TIPP/Investor's Business Daily/Christian Science Monitor | Okay, and generally speaking, how satisfied are you with the direction that the country is going in at this time in terms of morals and ethics? Would you say you are...very satisfied, somewhat satisfied, not very satisfied, or not at all satisfied? | 2002 | 902 |
| 167 | TIPP/Investor's Business Daily/Christian Science Monitor | Okay, and generally speaking, how satisfied are you with the direction that the country is going in at this time in terms of morals and ethics? Would you say you are...very satisfied, somewhat satisfied, not very satisfied, or not at all satisfied? | 2002 | 906 |
| 168 | TIPP/Investor's Business Daily/Christian Science Monitor | Okay, and generally speaking, how satisfied are you with the direction that the country is going in at this time in terms of morals and ethics? Would you say you are...very satisfied, somewhat satisfied, not very satisfied, or not at all satisfied? | 2001 | 921 |
| 169 | TIPP/Investor's Business Daily/Christian Science Monitor | Okay, and generally speaking, how satisfied are you with the direction that the country is going in at this time in terms of morals and ethics? Would you say you are...very satisfied, somewhat satisfied, not very satisfied, or not at all satisfied? | 2001 | 920 |
| 170 | TIPP/Investor's Business Daily/Christian Science Monitor | Okay, and generally speaking, how satisfied are you with the direction that the country is going in at this time in terms of morals and ethics? Would you say you are...very satisfied, somewhat satisfied, not very satisfied, or not at all satisfied? | 2001 | 925 |
| 171 | TIPP/Investor's Business Daily/Christian Science Monitor | Okay, and generally speaking, how satisfied are you with the direction that the country is going in at this time in terms of morals and ethics? Would you say you are...very satisfied, somewhat satisfied, not very satisfied, or not at all satisfied? | 2001 | 909 |
| 172 | TIPP/Investor's Business Daily/Christian Science Monitor | Okay, and generally speaking, how satisfied are you with the direction that the country is going in at this time in terms of morals and ethics? Would you say you are...very satisfied, somewhat satisfied, not very satisfied, or not at all satisfied? | 2001 | 949 |
| 173 | TIPP/Investor's Business Daily/Christian Science Monitor | Okay, and generally speaking, how satisfied are you with the direction that the country is going in at this time in terms of morals and ethics? Would you say you are...very satisfied, somewhat satisfied, not very satisfied, or not at all satisfied? | 2001 | 909 |
| 174 | TIPP/Investor's Business Daily/Christian Science Monitor | Okay, and generally speaking, how satisfied are you with the direction that the country is going in at this time in terms of morals and ethics? Would you say you are...very satisfied, somewhat satisfied, not very satisfied, or not at all satisfied? | 2001 | 909 |
| 175 | Economist - PTN | The government has reported a steady decline in the rate of violent crime over the last twenty years. Do you think the government is correct and the rate of violent crime has decreased or do you think the rate of violent crime has remained the same or even increased over the past twenty years? | 2019 | 1500 |
| 176 | Kaiser Family Foundation - PTN | For each issue I read please tell me if you think it is a major problem facing our country, a minor problem or not a problem at all. Decline in moral values. | 1999 | 3884 |
| 177 | YouGov | The government has reported a steady decline in the rate of violent crime over the last twenty years. Do you think the government is correct and the rate of violent crime has decreased or do you think the rate of violent crime has remained the same or even increased over the past twenty years? | 2014 | 1000 |

Table S2: Archival Survey Questions (non-US Sample), Study 1

| **#** | **Source** | **Question** | **Country** | **Year** | **N** |
| --- | --- | --- | --- | --- | --- |
| 1 | Gallup | Do you believe that society as a whole is less moral today than it was 50 years ago, or do you not believe that? | UK | 1996 | 1000 |
| 2 | Pew | Here is a list of things that may or may not be problems in our country. As I read each one, please tell me if you think it is a very big problem, a moderately big problem, a small problem, or not a problem at all. Moral decline. | Brazil | 2006 | 700 |
| 3 | Pew | Here is a list of things that may or may not be problems in our country. As I read each one, please tell me if you think it is a very big problem, a moderately big problem, a small problem, or not a problem at all. Moral decline. | Chile | 2006 | 600 |
| 4 | Pew | Here is a list of things that may or may not be problems in our country. As I read each one, please tell me if you think it is a very big problem, a moderately big problem, a small problem, or not a problem at all. Moral decline. | Guatemala | 2006 | 1005 |
| 5 | Pew | Here is a list of things that may or may not be problems in our country. As I read each one, please tell me if you think it is a very big problem, a moderately big problem, a small problem, or not a problem at all. Moral decline. | Kenya | 2006 | 655 |
| 6 | Pew | Here is a list of things that may or may not be problems in our country. As I read each one, please tell me if you think it is a very big problem, a moderately big problem, a small problem, or not a problem at all. Moral decline. | Nigeria | 2006 | 650 |
| 7 | Pew | Here is a list of things that may or may not be problems in our country. As I read each one, please tell me if you think it is a very big problem, a moderately big problem, a small problem, or not a problem at all. Moral decline. | South Africa | 2006 | 800 |
| 8 | Pew | Here is a list of things that may or may not be problems in our country. As I read each one, please tell me if you think it is a very big problem, a moderately big problem, a small problem, or not a problem at all. Moral decline. | India | 2006 | 726 |
| 9 | Pew | Here is a list of things that may or may not be problems in our country. As I read each one, please tell me if you think it is a very big problem, a moderately big problem, a small problem, or not a problem at all. Moral decline. | Philippines | 2006 | 1000 |
| 10 | Pew | Here is a list of things that may or may not be problems in our country. As I read each one, please tell me if you think it is a very big problem, a moderately big problem, a small problem, or not a problem at all. Moral decline. | South Korea | 2006 | 600 |
| 11 | YouGov | Compared with ten years ago, when Labour came to power, do you think Britain is a fairer society, or less fair, or has there been little change? | UK | 2007 | 589 |
| 12 | Eurobarometer | If you compare the situation with 5 years ago, would you say that the following types of discrimination are more common or less common in our country? Discrimination on the basis of ethnic origin. | EU | 2007 | 51718 |
| 13 | Eurobarometer | If you compare the situation with 5 years ago, would you say that the following types of discrimination are more common or less common in our country? Discrimination on the basis of gender. | EU | 2007 | 51718 |
| 14 | Eurobarometer | If you compare the situation with 5 years ago, would you say that the following types of discrimination are more common or less common in our country? Discrimination on the basis of sexual orientation. | EU | 2007 | 51718 |
| 15 | Eurobarometer | If you compare the situation with 5 years ago, would you say that the following types of discrimination are more common or less common in our country? Discrimination on the basis of age. | EU | 2007 | 51718 |
| 16 | Eurobarometer | If you compare the situation with 5 years ago, would you say that the following types of discrimination are more common or less common in our country? Discrimination on the basis of religion or beliefs. | EU | 2007 | 51718 |
| 17 | Eurobarometer | If you compare the situation with 5 years ago, would you say that the following types of discrimination are more common or less common in our country? Discrimination on the basis of disability. | EU | 2007 | 51718 |
| 18 | Market & Opinion Research International - PTN | I am going to read out some statements. I would like you to tell me how strongly you agree or disagree with each. Young people today do more for society and the community than their parents' generation did. | UK | 2000 | No information included |
| 19 | Pew | Here is a list of things that may be problems in our country. As I read each one, please tell me if you think it is a very big problem, a moderately big problem, a small problem, or not a problem at all...moral decline. | Angola | 2002 | 780 |
| 20 | Pew | Here is a list of things that may be problems in our country. As I read each one, please tell me if you think it is a very big problem, a moderately big problem, a small problem, or not a problem at all...moral decline. | Argentina | 2002 | 814 |
| 21 | Pew | Here is a list of things that may be problems in our country. As I read each one, please tell me if you think it is a very big problem, a moderately big problem, a small problem, or not a problem at all...moral decline. | Bangladesh | 2002 | 689 |
| 22 | Pew | Here is a list of things that may be problems in our country. As I read each one, please tell me if you think it is a very big problem, a moderately big problem, a small problem, or not a problem at all...moral decline. | Bolivia | 2002 | 782 |
| 23 | Pew | Here is a list of things that may be problems in our country. As I read each one, please tell me if you think it is a very big problem, a moderately big problem, a small problem, or not a problem at all...moral decline. | Brazil | 2002 | 1000 |
| 24 | Pew | Here is a list of things that may be problems in our country. As I read each one, please tell me if you think it is a very big problem, a moderately big problem, a small problem, or not a problem at all...moral decline. | Britain | 2002 | 501 |
| 25 | Pew | Here is a list of things that may be problems in our country. As I read each one, please tell me if you think it is a very big problem, a moderately big problem, a small problem, or not a problem at all...moral decline. | Bulgaria | 2002 | 514 |
| 26 | Pew | Here is a list of things that may be problems in our country. As I read each one, please tell me if you think it is a very big problem, a moderately big problem, a small problem, or not a problem at all...moral decline. | Canada | 2002 | 500 |
| 27 | Pew | Here is a list of things that may be problems in our country. As I read each one, please tell me if you think it is a very big problem, a moderately big problem, a small problem, or not a problem at all...moral decline. | Czech Republic | 2002 | 500 |
| 28 | Pew | Here is a list of things that may be problems in our country. As I read each one, please tell me if you think it is a very big problem, a moderately big problem, a small problem, or not a problem at all...moral decline. | France | 2002 | 507 |
| 29 | Pew | Here is a list of things that may be problems in our country. As I read each one, please tell me if you think it is a very big problem, a moderately big problem, a small problem, or not a problem at all...moral decline. | Germany | 2002 | 1000 |
| 30 | Pew | Here is a list of things that may be problems in our country. As I read each one, please tell me if you think it is a very big problem, a moderately big problem, a small problem, or not a problem at all...moral decline. | Ghana | 2002 | 702 |
| 31 | Pew | Here is a list of things that may be problems in our country. As I read each one, please tell me if you think it is a very big problem, a moderately big problem, a small problem, or not a problem at all...moral decline. | Guatemala | 2002 | 500 |
| 32 | Pew | Here is a list of things that may be problems in our country. As I read each one, please tell me if you think it is a very big problem, a moderately big problem, a small problem, or not a problem at all...moral decline. | Honduras | 2002 | 506 |
| 33 | Pew | Here is a list of things that may be problems in our country. As I read each one, please tell me if you think it is a very big problem, a moderately big problem, a small problem, or not a problem at all...moral decline. | India | 2002 | 2189 |
| 34 | Pew | Here is a list of things that may be problems in our country. As I read each one, please tell me if you think it is a very big problem, a moderately big problem, a small problem, or not a problem at all...moral decline. | Indonesia | 2002 | 1017 |
| 35 | Pew | Here is a list of things that may be problems in our country. As I read each one, please tell me if you think it is a very big problem, a moderately big problem, a small problem, or not a problem at all...moral decline. | Italy | 2002 | 5018 |
| 36 | Pew | Here is a list of things that may be problems in our country. As I read each one, please tell me if you think it is a very big problem, a moderately big problem, a small problem, or not a problem at all...moral decline. | Ivory Coast | 2002 | 708 |
| 37 | Pew | Here is a list of things that may be problems in our country. As I read each one, please tell me if you think it is a very big problem, a moderately big problem, a small problem, or not a problem at all...moral decline. | Japan | 2002 | 702 |
| 38 | Pew | Here is a list of things that may be problems in our country. As I read each one, please tell me if you think it is a very big problem, a moderately big problem, a small problem, or not a problem at all...moral decline. | Kenya | 2002 | 658 |
| 39 | Pew | Here is a list of things that may be problems in our country. As I read each one, please tell me if you think it is a very big problem, a moderately big problem, a small problem, or not a problem at all...moral decline. | Lebanon | 2002 | 1000 |
| 40 | Pew | Here is a list of things that may be problems in our country. As I read each one, please tell me if you think it is a very big problem, a moderately big problem, a small problem, or not a problem at all...moral decline. | Mali | 2002 | 697 |
| 41 | Pew | Here is a list of things that may be problems in our country. As I read each one, please tell me if you think it is a very big problem, a moderately big problem, a small problem, or not a problem at all...moral decline. | Mexico | 2002 | 996 |
| 42 | Pew | Here is a list of things that may be problems in our country. As I read each one, please tell me if you think it is a very big problem, a moderately big problem, a small problem, or not a problem at all...moral decline. | Nigeria | 2002 | 1000 |
| 43 | Pew | Here is a list of things that may be problems in our country. As I read each one, please tell me if you think it is a very big problem, a moderately big problem, a small problem, or not a problem at all...moral decline. | Pakistan | 2002 | 2032 |
| 44 | Pew | Here is a list of things that may be problems in our country. As I read each one, please tell me if you think it is a very big problem, a moderately big problem, a small problem, or not a problem at all...moral decline. | Peru | 2002 | 711 |
| 45 | Pew | Here is a list of things that may be problems in our country. As I read each one, please tell me if you think it is a very big problem, a moderately big problem, a small problem, or not a problem at all...moral decline. | Philippines | 2002 | 700 |
| 46 | Pew | Here is a list of things that may be problems in our country. As I read each one, please tell me if you think it is a very big problem, a moderately big problem, a small problem, or not a problem at all...moral decline. | Poland | 2002 | 500 |
| 47 | Pew | Here is a list of things that may be problems in our country. As I read each one, please tell me if you think it is a very big problem, a moderately big problem, a small problem, or not a problem at all...moral decline. | Russia | 2002 | 1002 |
| 48 | Pew | Here is a list of things that may be problems in our country. As I read each one, please tell me if you think it is a very big problem, a moderately big problem, a small problem, or not a problem at all...moral decline. | South Africa | 2002 | 700 |
| 49 | Pew | Here is a list of things that may be problems in our country. As I read each one, please tell me if you think it is a very big problem, a moderately big problem, a small problem, or not a problem at all...moral decline. | South Korea | 2002 | 719 |
| 50 | Pew | Here is a list of things that may be problems in our country. As I read each one, please tell me if you think it is a very big problem, a moderately big problem, a small problem, or not a problem at all...moral decline. | Senegal | 2002 | 710 |
| 51 | Pew | Here is a list of things that may be problems in our country. As I read each one, please tell me if you think it is a very big problem, a moderately big problem, a small problem, or not a problem at all...moral decline. | Slovakia | 2002 | 500 |
| 52 | Pew | Here is a list of things that may be problems in our country. As I read each one, please tell me if you think it is a very big problem, a moderately big problem, a small problem, or not a problem at all...moral decline. | Tanzania | 2002 | 720 |
| 53 | Pew | Here is a list of things that may be problems in our country. As I read each one, please tell me if you think it is a very big problem, a moderately big problem, a small problem, or not a problem at all...moral decline. | Turkey | 2002 | 1005 |
| 54 | Pew | Here is a list of things that may be problems in our country. As I read each one, please tell me if you think it is a very big problem, a moderately big problem, a small problem, or not a problem at all...moral decline. | Uganda | 2002 | 1008 |
| 55 | Pew | Here is a list of things that may be problems in our country. As I read each one, please tell me if you think it is a very big problem, a moderately big problem, a small problem, or not a problem at all...moral decline. | Ukraine | 2002 | 500 |
| 56 | Pew | Here is a list of things that may be problems in our country. As I read each one, please tell me if you think it is a very big problem, a moderately big problem, a small problem, or not a problem at all...moral decline. | Uzbekistan | 2002 | 700 |
| 57 | Pew | Here is a list of things that may be problems in our country. As I read each one, please tell me if you think it is a very big problem, a moderately big problem, a small problem, or not a problem at all...moral decline. | Venezuela | 2002 | 700 |
| 58 | ICM/Guardian | The Labour government came to power in 1997. With that in mind, do you agree or disagree with the following statement? People have become more selfish about money. | UK | 2006 | No information included |

Table S3: Archival Survey Questions (US Sample) and Results, Study 4

|  | **Source** | **Question** | **Response options** | **Years** | **N** | ***b*** | **R^2^** | **% of 89% HDI in ROPE** |
| --- | --- | --- | --- | --- | --- | --- | --- | --- |
| 1 | Gallup | How would you rate the overall state of moral values in this country today? | Excellent [4], Good [3], Only Fair [2], Poor [1] | 2002-2020 | 20,863 | -0.006 | 0.002 | 100 |
| 2 | Gallup | Next, we'd like to know how you feel about the state of the nation in each of the following areas. For each one, please say whether you are -- very satisfied, somewhat satisfied, somewhat dissatisfied or very dissatisfied. If you don't have enough information about a particular subject to rate it, just say so. How about -- the moral and ethical climate? | Very satisfied [4], Somewhat satisfied [3], Somewhat dissatisfied [2], Very dissatisfied [1] | 2001-2008, 2012-2020 | 17,015 | -0.01 | 0.008 | 100 |
| 3 | Gallup | Which of the following things, if any, have you, personally, done in the past 12 months? How about Donated money to any other charitable cause | Yes [1], No [0] | 2001, 2003, 2005, 2008, 2009a, 2009b, 2013, 2017, 2020 | 9,222 | -0.02 | 0.002 | 100 |
| 4 | Gallup | Which of the following things, if any, have you, personally, done in the past 12 months? Given blood | Yes [1], No [0] | 2001, 2003, 2005, 2008, 2013, 2017, 2020 | 7,131 | -0.01 | 0.001 | 100 |
| 5 | Gallup | Which of the following things, if any, have you, personally, done in the past 12 months? Volunteered your time to any other charitable cause | Yes [1], No [0] | 2001, 2003, 2005, 2008, 2009a, 2009b, 2013, 2017, 2020 | 9,222 | 0.006 | 0.0003 | 100 |
| 6 | Gallup | Does your supervisor always create an environment that is trusting and open, or not? (asked only of those with a job and a supervisor) | Yes [1], No [0] | 2008, 2009, 2010, 2011, 2012, 2013, 2014 | 957,762 | 0.02 | 0.0001 | 100 |
| 7 | Gallup | Do you feel safe walking alone at night in the city or area where you live? | Yes [1], No [0] | 2008, 2009, 2010, 2011, 2012, 2013 | 1,774,388 | 0.01 | 0.00005 | 100 |
| 8 | Gallup | On a five-point scale, where 5 means strongly agree and 1 means strongly disagree, please rate your level of agreement with the following items. You always feel safe and secure. | 1 [strongly disagree] - 5 [strongly agree] | 2014-2017 | 691,671 | 0.001 | 0.000001 | 100 |
| 9 | Gallup | Have you done any of the following in the past month? How about volunteered your time to an organization? | Yes [1], No [0] | 2007-2019 | 12,126 | -0.02 | 0.001 | 100 |
| 10 | Gallup | Within the last 12 months, have you had money or property stolen from you or another household member? | Yes [1], No [0] | 2006-2019 | 12,561 | 0.002 | 0.000007 | 100 |
| 11 | Gallup | Now, please think about yesterday, from the morning until the end of the day. Think about where you were, what you were doing, who you were with, and how you felt. Were you treated with respect all day yesterday? | Yes [1], No [0] | 2006-2019 | 14,255 | 0.009 | 0.0002 | 100 |
| 12 | Gallup | Do you feel safe walking alone at night in the city or area where you live? | Yes [1], No [0] | 2006-2019 | 12,505 | -0.02 | 0.0007 | 100 |
| 13 | Gallup | If you were in trouble, do you have relatives or friends you can count on to help you whenever you need them, or not? | Yes [1], No [0] | 2006, 2008-2019 | 12,935 | -0.04 | 0.004 | 100 |
| 14 | Gallup | Please tell me which, if any, of these incidents have happened to you or your household within the last twelve months? Your house or apartment broken into. | Yes [1], No [0] | 2000-2019 | 19,348 | -0.004 | 0.00006 | 100 |
| 15 | Gallup | Within the past 12 months, have you been assaulted or mugged? | Yes [1], No [0] | 2015-2019 | 4,118 | -0.11 | 0.002 | 87.59 |
| 16 | Gallup | Next, I'm going to read some things people do because of their concern over crime. Please tell me which, if any, of these things you, yourself, do or have done. Keep a dog for protection. | Yes [1], No [0] | 2000, 2001, 2002, 2003, 2005, 2007 | 6,064 | -0.004 | 0.00002 | 100 |
| 17 | Gallup | Have you done any of the following in the past month? How about helped a stranger or someone you didn't know who needed help? | Yes [1], No [0] | 2007-2019 | 12,126 | 0.0009 | 0.000002 | 100 |
| 18 | Gallup | Next, I'm going to read some things people do because of their concern over crime. Please tell me which, if any, of these things you, yourself, do or have done. Avoid going to certain places or neighborhoods you might otherwise want to go to. | Yes [1], No [0] | 2000, 2001, 2002, 2003, 2005, 2007 | 6,064 | -0.01 | 0.0002 | 100 |
| 19 | Gallup | Next, I'm going to read some things people do because of their concern over crime. Please tell me which, if any, of these things you, yourself, do or have done. Carry mace or pepper spray. | Yes [1], No [0] | 2000, 2001, 2002, 2003, 2005, 2007 | 6,064 | -0.03 | 0.0006 | 100 |
| 20 | Gallup | Next, I'm going to read some things people do because of their concern over crime. Please tell me which, if any, of these things you, yourself, do or have done. Had a burglar alarm installed in your home. | Yes [1], No [0] | 2000, 2001, 2002, 2003, 2005, 2007 | 6,064 | 0.06 | 0.004 | 100 |
| 21 | Gallup | Next, I'm going to read some things people do because of their concern over crime. Please tell me which, if any, of these things you, yourself, do or have done. Carry a knife for defense. | Yes [1], No [0] | 2000, 2001, 2002, 2003, 2005, 2007 | 6,064 | 0.03 | 0.001 | 100 |
| 22 | Gallup | Next, I'm going to read some things people do because of their concern over crime. Please tell me which, if any, of these things you, yourself, do or have done. Carry a gun for defense. | Yes [1], No [0] | 2000, 2001, 2002, 2003, 2005, 2007 | 6,064 | 0.005 | 0.00002 | 100 |
| 23 | Gallup | Next, I'm going to read some things people do because of their concern over crime. Please tell me which, if any, of these things you, yourself, do or have done. Bought a gun for protection of yourself or your home. | Yes [1], No [0] | 2000, 2001, 2002, 2003, 2005, 2007 | 6,064 | -0.004 | 0.00002 | 100 |
| 24 | Gallup | Have you, personally, EVER been the victim of a crime where you were physically harmed, or threatened with physical harm? | Yes [1], No [0] | 2000, 2011 | 2,024 | -0.02 | 0.001 | 100 |
| 25 | Gallup | Next, I'm going to read a list of problems facing the country. For each one, please tell me if you personally worry about this problem a great deal, a fair amount, only a little or not at all? First, how much do you personally worry about crime and violence? | Great deal [4], fair amount [3], only a little [2], not at all [1] | 2001-2008, 2010-2020 | 18,789 | -0.005 | 0.001 | 100 |
| 26 | Gallup | Please tell me which, if any, of these incidents have happened to you or your household within the last twelve months? You or another household member had your computer or smartphone hacked and the information stolen by unauthorized persons. | Yes [1], No [0] | 2003-2011, 2014 | 10,154 | 0.06 | 0.005 | 100 |
| 27 | Gallup | Please tell me which, if any, of these incidents have happened to you or your household within the last twelve months? You or another household member had information from a credit card used at a store stolen by computer hackers. | Yes [1], No [0] | 2014-2016 | 3,049 | -0.007 | 0.000005 | 100 |
| 28 | Gallup | Please tell me which, if any, of these incidents have happened to you or your household within the last twelve months? You or another household member had personal, credit card or financial information stolen by computer hackers | Yes [1], No [0] | 2017-2019 | 3,089 | -0.05 | 0.0003 | 100 |
| 29 | Gallup | Please tell me which, if any, of these incidents have happened to you or your household within the last twelve months? You or another household member was the victim of identity theft. | Yes [1], No [0] | 2009, 2010, 2011, 2013, 2015-2019 | 9,212 | 0.06 | 0.007 | 100 |
| 30 | Gallup | Please tell me which, if any, of these incidents have happened to you or your household within the last twelve months? You or another household member was sexually assaulted. | Yes [1], No [0] | 2000, 2002-2011, 2013-2019 | 18,337 | 0.01 | 0.0003 | 100 |
| 31 | Gallup | Please tell me which, if any, of these incidents have happened to you or your household within the last twelve months? You or another household member mugged or physically assaulted. | Yes [1], No [0] | 2000-2011, 2013-2019 | 19,348 | -0.008 | 0.0003 | 100 |
| 32 | Gallup | Please tell me which, if any, of these incidents have happened to you or your household within the last twelve months? Money or property taken from you or another household member by force, with gun, knife, weapon or physical attack, or by threat of force. | Yes [1], No [0] | 2000-2011, 2013-2019 | 19,348 | -0.002 | 0.00001 | 100 |
| 33 | Gallup | Please tell me which, if any, of these incidents have happened to you or your household within the last twelve months? A home, car, or property owned by you or another household member vandalized. | Yes [1], No [0] | 2000-2011, 2013-2019 | 19,348 | -0.008 | 0.0003 | 100 |
| 34 | Gallup | Please tell me which, if any, of these incidents have happened to you or your household within the last twelve months? A car owned by you or another household member stolen. | Yes [1], No [0] | 2000-2011, 2013-2019 | 19,348 | -0.02 | 0.001 | 100 |
| 35 | Gallup | Please tell me which, if any, of these incidents have happened to you or your household within the last twelve months? Money or property stolen from you or another member of your household. | Yes [1], No [0] | 2000-2011, 2013-2019 | 19,348 | 0.006 | 0.0002 | 100 |
| 36 | Gallup | How often do you, yourself, worry about the following things -- frequently, occasionally, rarely or never? How about -- Having your email, passwords or electronic records hacked into? | Frequently [4], Occasionally [3], Rarely [2], Never [1] | 2014, 2016 | 2,065 | 0.01 | 0.0001 | 100 |
| 37 | Gallup | How often do you, yourself, worry about the following things -- frequently, occasionally, rarely or never? How about -- Having the credit card information you have used at stores stolen by computer hackers? | Frequently [4], Occasionally [3], Rarely [2], Never [1] | 2014-2016 | 3,080 | -0.02 | 0.0001 | 100 |
| 38 | Gallup | How often do you, yourself, worry about the following things -- frequently, occasionally, rarely or never? How about -- Having your personal, credit card, or financial information stolen by computer hackers? | Frequently [4], Occasionally [3], Rarely [2], Never [1] | 2017-2019 | 3,089 | 0.04 | 0.001 | 100 |
| 39 | Gallup | How often do you, yourself, worry about the following things -- frequently, occasionally, rarely or never? How about -- Being a victim of identity theft? | Frequently [4], Occasionally [3], Rarely [2], Never [1] | 2009, 2010, 2011, 2013, 2015-2019 | 9,212 | 0.003 | 0.00007 | 100 |
| 40 | Gallup | How often do you, yourself, worry about the following things -- frequently, occasionally, rarely or never? How about -- Being a victim of terrorism? | Frequently [4], Occasionally [3], Rarely [2], Never [1] | 2001-2011, 2013-2019 | 18,336 | -0.02 | 0.01 | 100 |
| 41 | Gallup | How often do you, yourself, worry about the following things -- frequently, occasionally, rarely or never? How about -- Being the victim of a hate crime? | Frequently [4], Occasionally [3], Rarely [2], Never [1] | 2000, 2001, 2003-2011, 2013-2019 | 18,346 | 0.01 | 0.005 | 100 |
| 42 | Gallup | How often do you, yourself, worry about the following things -- frequently, occasionally, rarely or never? How about -- Being attacked while driving your car? | Frequently [4], Occasionally [3], Rarely [2], Never [1] | 2000, 2001, 2003-2011, 2013-2019 | 18,346 | -0.008 | 0.002 | 100 |
| 43 | Gallup | How often do you, yourself, worry about the following things -- frequently, occasionally, rarely or never? How about -- Having a school-aged child of yours physically harmed while attending school? | Frequently [4], Occasionally [3], Rarely [2], Never [1] | 2000-2011, 2013-2019 | 19,348 | -0.005 | 0.0008 | 100 |
| 44 | Gallup | How often do you, yourself, worry about the following things -- frequently, occasionally, rarely or never? How about -- Being assaulted or killed by a coworker or other employee where you work? | Frequently [4], Occasionally [3], Rarely [2], Never [1] | 2000-2011, 2013-2019 | 19,348 | 0.001 | 0.0001 | 100 |
| 45 | Gallup | How often do you, yourself, worry about the following things -- frequently, occasionally, rarely or never? How about -- Having your car stolen or broken into? | Frequently [4], Occasionally [3], Rarely [2], Never [1] | 2000-2011, 2013-2019 | 19,348 | -0.005 | 0.0007 | 100 |
| 46 | Gallup | How often do you, yourself, worry about the following things -- frequently, occasionally, rarely or never? How about -- Being sexually assaulted? | Frequently [4], Occasionally [3], Rarely [2], Never [1] | 2000-2011, 2013-2019 | 19,796 | -0.0004 | 0.000005 | 100 |
| 47 | Gallup | How often do you, yourself, worry about the following things -- frequently, occasionally, rarely or never? How about -- Your home being burglarized when you are not there? | Frequently [4], Occasionally [3], Rarely [2], Never [1] | 2000-2011, 2013-2019 | 19,796 | -0.004 | 0.0006 | 100 |
| 48 | Gallup | How often do you, yourself, worry about the following things -- frequently, occasionally, rarely or never? How about -- Your home being burglarized when you are there? | Frequently [4], Occasionally [3], Rarely [2], Never [1] | 2000-2011, 2013-2019 | 19,796 | -0.002 | 0.00008 | 100 |
| 49 | Gallup | How often do you, yourself, worry about the following things -- frequently, occasionally, rarely or never? How about -- Getting murdered? | Frequently [4], Occasionally [3], Rarely [2], Never [1] | 2000-2011, 2013-2019 | 19,796 | 0.004 | 0.0006 | 100 |
| 50 | Gallup | How often do you, yourself, worry about the following things -- frequently, occasionally, rarely or never? How about -- Getting mugged? | Frequently [4], Occasionally [3], Rarely [2], Never [1] | 2000-2011, 2013-2019 | 19,796 | 0.001 | 0.00007 | 100 |
| 51 | Gallup | Overall, how would you describe the problem of crime in the area where you live -- is it extremely serious, very serious, moderately serious, not too serious or not serious at all? | Extremely [5]. Very [4], Somewhat [3], Not too [2], Not at all [1] | 2000, 2003-2011, 2013-2019 | 17,819 | 0.002 | 0.0001 | 100 |
| 52 | Gallup | Overall, how would you describe the problem of crime in the United States -- is it extremely serious, very serious, moderately serious, not too serious or not serious at all? | Extremely [5]. Very [4], Somewhat [3], Not too [2], Not at all [1] | 2000, 2003-2011, 2013-2019 | 18,025 | -0.0002 | 0.000002 | 100 |
| 53 | Gallup | Is there any area near where you live -- that is, within a mile -- where you would be afraid to walk alone at night? | Yes [1], No [0] | 1965, 1967, 1968, 1972, 1975, 1977, 1979, 1981, 1982, 1983, 1989, 1990, 1992, 1993, 1994, 1996, 1997, 2000-2011, 2013-2019 | 37,882 | -0.006 | 0.002 | 100 |
| 54 | General Social Survey | Now I''m going to read you a list of statements that might or might not describe your main job. Please tell me whether you strongly agree, agree, disagree, or strongly disagree with each of these statements. I. At the place where I work, I am treated with respect | Strongly agree [5], Agree [4], Neither agree nor disagree [3], Disagree [2], Strongly disagree [1] | 2002, 2006, 2010, 2014, 2018 | 7,301 | -0.004 | 0.001 | 100 |
| 55 | General Social Survey | Would you say that most of the time people try to be helpful, or that they are mostly just looking out for themselves? | Helpful [1], Depends [2], Looking out for self [3] | 1972, 1973, 1975, 1976, 1978, 1980, 1983, 1984, 1986, 1987, 1988, 1990, 1991, 1993, 1994, 1996, 1998, 2000, 2002, 2004, 2006, 2008, 2010, 2012, 2014, 2016, 2018 | 41,009 | 0.002 | 0.0006 | 100 |
| 56 | General Social Survey | Please tell me whether you strongly agree, agree, neither agree nor disagree, disagree, or strongly disagree with the following statements: A. People should be willing to help others who are less fortunate | Strongly agree [5], Agree [4], Neither agree nor disagree [3], Disagree [2], Strongly disagree [1] | 2002, 2004, 2012, 2014 | 5,248 | 0.001 | 0.00006 | 100 |
| 57 | General Social Survey | Now I''m going to read you another list of statements about your main job. For each, please tell me if the statement is very true, somewhat true, not too true, or not at all true with respect to the work you do. N. The people I work with can be relied on when I need help | Very true [4], Somewhat true [3], Not too true [2], Not at all true [1] | 2002, 2006, 2010, 2014, 2018 | 7,235 | -0.001 | 0.0001 | 100 |
| 58 | General Social Survey | During the past 12 months, how often have you done each of the following things: D. Allowed a stranger to go ahead of you in line | More than once a week [6], Once a week [5], Once a month [4], At least 2-3 times in the past year [3], Once in the past year [2], Not at all [1] | 2002, 2004, 2012, 2014 | 4,916 | 0.003 | 0.0002 | 100 |
| 59 | General Social Survey | During the past 12 months, how often have you done each of the following things: G. Offered your seat on a bus or in a public place to a stranger who was standing | More than once a week [6], Once a week [5], Once a month [4], At least 2-3 times in the past year [3], Once in the past year [2], Not at all [1] | 2002, 2004, 2012, 2014 | 5,138 | 0.0006 | 0.000006 | 100 |
| 60 | General Social Survey | During the past 12 months, how often have you done each of the following things: I. Carried a stranger's belongings, like groceries, a suitcase, or shopping bag | More than once a week [6], Once a week [5], Once a month [4], At least 2-3 times in the past year [3], Once in the past year [2], Not at all [1] | 2002, 2004, 2012, 2014 | 5,157 | 0.003 | 0.0001 | 100 |
| 61 | General Social Survey | During the past 12 months, how often have you done each of the following things: J. Given directions to a stranger | More than once a week [6], Once a week [5], Once a month [4], At least 2-3 times in the past year [3], Once in the past year [2], Not at all [1] | 2002, 2004, 2012, 2014 | 4,978 | -0.001 | 0.003 | 100 |
| 62 | General Social Survey | During the past 12 months, how often have you done each of the following things: A. Donated blood | More than once a week [6], Once a week [5], Once a month [4], At least 2-3 times in the past year [3], Once in the past year [2], Not at all [1] | 2002, 2004, 2012, 2014 | 5,257 | -0.01 | 0.004 | 100 |
| 63 | General Social Survey | During the past 12 months, how often have you done each of the following things: B. Given food or money to a homeless person | More than once a week [6], Once a week [5], Once a month [4], At least 2-3 times in the past year [3], Once in the past year [2], Not at all [1] | 2002, 2004, 2012, 2014 | 5,091 | 0.01 | 0.002 | 100 |
| 64 | General Social Survey | During the past 12 months, how often have you done each of the following things: C. Returned money to a cashier after getting too much change | More than once a week [6], Once a week [5], Once a month [4], At least 2-3 times in the past year [3], Once in the past year [2], Not at all [1] | 2002, 2004, 2012, 2014 | 5,181 | -0.02 | 0.01 | 100 |
| 65 | General Social Survey | During the past 12 months, how often have you done each of the following things: E. Done volunteer work for a charity | More than once a week [6], Once a week [5], Once a month [4], At least 2-3 times in the past year [3], Once in the past year [2], Not at all [1] | 2002, 2004, 2012, 2014 | 5,084 | 0.005 | 0.0003 | 100 |
| 66 | General Social Survey | During the past 12 months, how often have you done any of the following things for people you know personally, such as relatives, friends, neighbors or other acquaintances?  B. Lent quite a bit of money to another person | More than once a week [6], Once a week [5], Once a month [4], At least 2-3 times in the past year [3], Once in the past year [2], Not at all [1] | 2002, 2004, 2012, 2014 | 4,041 | -0.02 | 0.005 | 100 |
| 67 | General Social Survey | During the past 12 months, how often have you done each of the following things: K. Let someone you didn''t know well borrow a item of some value like dishes or tools. | More than once a week [6], Once a week [5], Once a month [4], At least 2-3 times in the past year [3], Once in the past year [2], Not at all [1] | 2002, 2004, 2012, 2014 | 5,198 | -0.004 | 0.0003 | 100 |
| 68 | General Social Survey | During the past 12 months, how often have you done each of the following things: H. Looked after a person's plants, mail, or pets while they were away | More than once a week [6], Once a week [5], Once a month [4], At least 2-3 times in the past year [3], Once in the past year [2], Not at all [1] | 2002, 2004, 2012, 2014 | 5,149 | -0.01 | 0.002 | 100 |
| 69 | General Social Survey | The following are things that you may experience in your daily life. Please tell me how often these occur. A. I feel a selfless caring for others. | Many times a day [6], Every day [5], Most days [4], Some days [3], Once in a while [2], Never or almost never [1] | 2002, 2004, 2012, 2014 | 5,151 | -0.0002 | 0.0000004 | 100 |
| 70 | General Social Survey | Please tell me whether you strongly agree, agree, neither agree nor disagree, disagree, or strongly disagree with the following statements: C. Personally assisting people in trouble is very important to me | Strongly agree [5], Agree [4], Neither agree nor disagree [3], Disagree [2], Strongly disagree [1] | 2002, 2004, 2012, 2014 | 5,247 | 0.005 | 0.001 | 100 |
| 71 | General Social Survey | Is there any area right around here--that is, within a mile--where you would be afraid to walk alone at night? | Yes [1], No [0] | 1973, 1974, 1976, 1977, 1980, 1982, 1984, 1985, 1987, 1988, 1990, 1991, 1993, 1994, 1996, 1998, 2000, 2002, 2004, 2006, 2008, 2010, 2012, 2014, 2016, 2018 | 39,441 | -0.01 | 0.005 | 100 |
| 72 | General Social Survey | Other people take credit for my work or ideas. | Often [4], Sometimes [3], Rarely [2], Never [1] | 2004, 2012 | 2,931 | 0.03 | 0.008 | 100 |
| 73 | General Social Survey | D. People at work treat me in a manner that puts me down or address me in unprofessional terms, either publicly or privately. | Often [4], Sometimes [3], Rarely [2], Never [1] | 2004, 2012 | 2,937 | 0.006 | 0.0008 | 100 |
| 74 | General Social Survey | H. People at work throw things, slam doors, or hit objects when they are upset with me. | Often [4], Sometimes [3], Rarely [2], Never [1] | 2004, 2012 | 2,942 | -0.003 | 0.0003 | 100 |
| 75 | General Social Survey | I. People at work shout or yell at me in a hostile manner. | Often [4], Sometimes [3], Rarely [2], Never [1] | 2004, 2012 | 2,940 | 0.006 | 0.001 | 100 |
| 76 | General Social Survey | M. At work, people are treated with respect. | Strongly agree [4], Agree [3], Disagree [2], Strongly disagree [1] | 2004, 2012 | 2,939 | -0.006 | 0.001 | 100 |
| 77 | General Social Survey | N. In my workplace, people "look the other way" when others are threatened, intimidated, or put down. | Strongly agree [4], Agree [3], Disagree [2], Strongly disagree [1] | 2004, 2012 | 2,887 | 0.06 | 0.09 | 100 |
| 78 | General Social Survey | Generally speaking, would you say that people can be trusted or that you can't be too careful in dealing with people? | Can trust [3], Depends [2], Can't be too careful [1] | 1972, 1973, 1975, 1976, 1980, 1983, 1984, 1986, 1987, 1988, 1990, 1991, 1993, 1994, 1996, 1998, 2000, 2002, 2004, 2006, 2008, 2010, 2012, 2014 | 41,258 | -0.006 | 0.008 | 100 |
| 79 | General Social Survey | Now, I'm going to ask you about various events and conditions that happen to people. I'm interested in those that happened to you during the last 12 months, that is since (CURRENT MONTH), (1990/2003). As I ask you about the specific events, please think carefully, so I can record things accurately. F. Next, did any of the following criminal or legal events occur to you since (CURRENT MONTH), (1990/2003)... 1. A robbery (e.g. a mugging or stick-up). | 1 = yes, 2 = no | 1991, 2004 | 2,341 | 0.0002 | -0.0004 | 100 |
| 80 | General Social Survey | During the past 12 months, how often have you done any of the following things for people you know personally, such as relatives, friends, neighbors or other acquaintances?  A. Helped someone outside of your household with housework or shopping | More than once a week  [6], Once a week [5], Once a month [4], At least two or three times in the past year [3], Once in the past year [2], Not at all in the past year [1] | 2002, 2004, 2012, 2014 | 5,042 | -0.05 | 0.03 | 100 |
| 81 | General Social Survey | During the past 12 months, how often have you done any of the following things for people you know personally, such as relatives, friends, neighbors or other acquaintances? C. Spent time talking with someone who was a bit down or depressed | More than once a week  [6], Once a week [5], Once a month [4], At least two or three times in the past year [3], Once in the past year [2], Not at all in the past year [1] | 2002, 2004, 2012, 2014 | 5,043 | -0.02 | 0.004 | 100 |
| 82 | General Social Survey | During the past 12 months, how often have you done any of the following things for people you know personally, such as relatives, friends, neighbors or other acquaintances?  D. Helped somebody to find a job | More than once a week  [6], Once a week [5], Once a month [4], At least two or three times in the past year [3], Once in the past year [2], Not at all in the past year [1] | 2002, 2004, 2012, 2014 | 5,047 | 0.001 | 0.00003 | 100 |
| 83 | General Social Survey | There are different opinions as to what it takes to be a good citizen. As far as you are concerned personally on a scale of 1 to 7, where 1 is not at all important and 7 is very important, how important is it‚Ä¶  H. To help people in America who are worse off than yourself | 1 [not at all important] - 7 [very important] | 2004, 2014 | 2,712 | -0.02 | 0.005 | 100 |
| 84 | General Social Survey | There are different opinions as to what it takes to be a good citizen. As far as you are concerned personally on a scale of 1 to 7, where 1 is not at all important and 7 is very important, how important is it‚Ä¶  I. To help people in the rest of the world who are worse off than yourself | 1 [not at all important] - 7 [very important] | 2004, 2014 | 2,689 | -0.02 | 0.003 | 100 |
| 85 | General Social Survey | During the past 12 months, how often have you done each of the following things:  F. Given money to a charity | More than once a week  [6], Once a week [5], Once a month [4], At least two or three times in the past year [3], Once in the past year [2], Not at all in the past year [1] | 2002, 2004, 2012, 2014 | 3,568 | -0.02 | 0.005 | 100 |
| 86 | KRC Research | Generally speaking, do you believe there is a major problem, minor problem, or not much of a problem with the general tone and level of civility in the country today? | Major problem [3], Minor problem [2], Not much of a problem [1] | 2010-2017, 2019 | 9192 | 0.006 | 0.0008 | 100 |
| 87 | KRC Research | In an average 7-day week, how many times would you say you encounter incivility, in your in-person interactions with people and online in social networks [2016-2019: "and in your online interactions with people]? Please use your best estimate. IN-PERSON INTERACTIONS. | 0, 1-5, 6-10, 11-15, 16+ | 2013-2017, 2019 | 5628 | -0.13 | 0.005 | 100 |
| 88 | KRC Research | In an average 7-day week, how many times would you say you encounter incivility, in your in-person interactions with people and online in social networks [2016-2019: "and in your online interactions with people]? Please use your best estimate. ONLINE INTERACTIONS. | 0, 1-5, 6-10, 11-15, 16+ | 2013-2017, 2019 | 5628 | 0.03 | 0.0002 | 100 |
| 89 | KRC Research | [ASKED OF THOSE EMPLOYED] How would you describe the general tone and level of civility in your place of employment? | Very civil [4], Somewhat civil [3], Somewhat uncivil [2], Very uncivil [1] | 2016, 2017, 2019 | 1606 | -0.002 | 0.00001 | 100 |
| 90 | KRC Research | Which of the following, if any, have you personally experienced? [instances of incivility] | Any [1], None [0] | 2011-2016 | 6,134 | -0.04 | 0.0007 | 100 |
| 91 | World Values Survey | Generally speaking, would you say that most people can be trusted or that you need to be very careful in dealing with people? | Can't be too careful [1], Most people can be trusted [0] | 1981, 1995, 1999, 2001, 2006, 2011, 2017 | 8,902 | 0.003 | 0.003 | 100 |
| 92 | World Values Survey | Here is a list of qualities that children can be encouraged to learn at home. Which, if any, do you consider to be especially important? Please choose up to five. UNSELFISHNESS | Not mentioned [0], Mentioned as important [1] | 1981, 1995, 1999, 2001, 2006, 2011, 2017 | 14,154 | 0.002 | 0.001 | 100 |
| 93 | World Values Survey | How much respect is there for individual human rights nowadays (in our country)?. Do you feel there is: | A great deal of respect for individual human rights [1], Fairly much respect [2], Not much respect [3], No respect at all [4] | 1999, 2001, 2006, 2011, 2017 | 8,304 | 0.02 | 0.005 | 100 |
| 94 | World Values Survey | Here is a list of qualities that children can be encouraged to learn at home. Which, if any, do you consider to be especially important? Please choose up to five. TOLERANCE | Not mentioned [0], Mentioned as important [1] | 1981, 1995, 1999, 2001, 2006, 2011, 2017 | 14,154 | 0.004 | 0.004 | 100 |
| 95 | World Values Survey | I'd like to ask you how much you trust people from various groups. Could you tell me for each whether you trust people from this group completely, somewhat, not very much or not at all? People of another religion. | Trust completely [1], Trust somewhat [2], Don't trust very much [3], Do not trust at all [4] | 2006, 2011, 2017 | 5932 | 0.02 | 0.001 | 100 |
| 96 | World Values Survey | I'd like to ask you how much you trust people from various groups. Could you tell me for each whether you trust people from this group completely, somewhat, not very much or not at all? People of another nationality. | Trust completely [1], Trust somewhat [2], Don't trust very much [3], Do not trust at all [4] | 2006, 2011, 2017 | 5921 | -0.006 | 0.0002 | 100 |
| 97 | World Values Survey | I'd like to ask you how much you trust people from various groups. Could you tell me for each whether you trust people from this group completely, somewhat, not very much or not at all? People you know personally. | Trust completely [1], Trust somewhat [2], Don't trust very much [3], Do not trust at all [4] | 2006, 2011, 2017 | 5958 | 0.003 | 0.00009 | 100 |
| 98 | World Values Survey | I'd like to ask you how much you trust people from various groups. Could you tell me for each whether you trust people from this group completely, somewhat, not very much or not at all? People you meet for the first time. | Trust completely [1], Trust somewhat [2], Don't trust very much [3], Do not trust at all [4] | 2006, 2011, 2017 | 5971 | 0.002 | 0.00001 | 100 |
| 99 | World Values Survey | I'd like to ask you how much you trust people from various groups. Could you tell me for each whether you trust people from this group completely, somewhat, not very much or not at all? Your neighborhood. | Trust completely [1], Trust somewhat [2], Don't trust very much [3], Do not trust at all [4] | 2006, 2011, 2017 | 5965 | 0.008 | 0.0003 | 100 |
| 100 | World Values Survey | I'd like to ask you how much you trust people from various groups. Could you tell me for each whether you trust people from this group completely, somewhat, not very much or not at all? Your family. | Trust completely [1], Trust somewhat [2], Don't trust very much [3], Do not trust at all [4] | 2006, 2011, 2017 | 5978 | 0.02 | 0.003 | 100 |
| 101 | World Values Survey | Do you think most people would try to take advantage of you if they got a chance, or would they try to be fair? Please show your response on this card, where 1 means that ‚Äúpeople would try to take advantage of you,‚Äù and 10 means that ‚Äúpeople would try to be fair‚Äù: | Most people try to take advantage of me [1] - Most people try to be fair [10] | 2006, 2011 | 3436 | 0.001 | 0.00003 | 100 |
| 102 | European Values Survey | Here is a list of qualities which children can be encouraged to learn at home. Which, if any, do you consider to be especially important? Please choose up to five. Tolerance and respect for other people | [participants could choose the quality from the list or not] | 1981-2017 (but varies by country) | 4164 | .02 | 0.02 | 100 |
| 103 | European Values Survey | Here is a list of qualities which children can be encouraged to learn at home. Which, if any, do you consider to be especially important? Please choose up to five. Unselfishness | [participants could choose the quality from the list or not] | 1981-2017 (but varies by country) | 4164 | .02 | 0.02 | 100 |
| 104 | European Values Survey | Please look carefully at the following list of voluntary organisations and activities and say ... b) which, if any, are you currently doing unpaid voluntary work for? Social welfare services for elderly, handicapped or deprived people | [participants could indicate they currently do unpaid work, or not] | 1981-2017 (but varies by country) | 4164 | -0.0008 | -0.00006 | 100 |
| 105 | European Values Survey | Please look carefully at the following list of voluntary organisations and activities and say ... b) which, if any, are you currently doing unpaid voluntary work for? And do you currently do any unpaid work for any of them? Third world development or human rights | [participants could indicate they currently do unpaid work, or not] | 1981-2017 (but varies by country) | 4164 | -0.001 | 0.001 | 100 |
| 106 | European Values Survey | Generally speaking, would you say that most people can be trusted or that you can't be too careful in dealing with people? | Most people can be trusted (recoded as 1), can't be too careful (recoded as 0) | 1981-2017 (but varies by country) | 4041 | 0.01 | 0.008 | 100 |
| 107 | European Values Survey | Here are some aspects of a job that people say are important. Please look at them and tell me which ones you personally think are important in a job? A useful job for society | [participants could choose the quality from the list or not] | 1981-2017 (but varies by country) | 4164 | -0.002 | -0.0001 | 100 |

Table S4: Archival Survey Questions (non-US Sample) and Results, Study 4

|  | **Source** | **Question** | **Response options** | **Years** | **N** | ***B*** | **R^2^** | **% of 89% HDI in ROPE** |
| --- | --- | --- | --- | --- | --- | --- | --- | --- |
| 1 | World Values Survey | And for which, if any, are you currently doing unpaid voluntary work? Unpaid work social welfare service for elderly, handicapped or deprived people | [mentioned or not] | 1981-2018 (but varies by country) | 49958 | 0.007 | 0.008 | 100 |
| 2 | World Values Survey | Here are some more aspects of a job that people say are important. Please look at them and tell me which ones you personally think are important in a job? Useful for society | [mentioned or not] | 1981-2018 (but varies by country) | 21905 | 0.05 | 0.001 | 37% |
| 3 | World Values Survey | Here is a list of qualities that children can be encouraged to learn at home. Which, if any, do you consider to be especially important? Please choose up to five. Not being selfish (unselfishness) | [mentioned or not] | 1981-2018 (but varies by country) | 404605 | 0.003 | 0.003 | 100 |
| 4 | World Values Survey | How much respect is there for individual human rights nowadays in this country? | 1 = a lot, 4 = no respect at all | 1981-2018 (but varies by country) | 288573 | -0.006 | 0.0009 | 100 |
| 5 | World Values Survey | Now I will briefly describe some people. Using this card, would you please indicate for each description whether that person is very much like you, like you, somewhat like you, not like you, or not at all like you? : "It is important to help people living nearby; to care for their needs" | 1 = not at all like me, 6 = very much like me | 1981-2018 (but varies by country) | 111196 | -0.02 | 0.001 | 100 |
| 6 | World Values Survey | Here is a list of qualities that children can be encouraged to learn at home. Which, if any, do you consider to be especially important? Please choose up to five. Tolerance and respect for other people | [mentioned or not] | 1981-2018 (but varies by country) | 408509 | 0.003 | 0.002 | 100 |
| 7 | World Values Survey | I‘d like to ask you how much you trust people from various groups. Could you tell me for each whether you trust people from this group completely, somewhat, not very much or not at all? People of another religion | 1 = trust completely, 4 = do not trust at all | 1981-2018 (but varies by country) | 212738 | -0.003 | 0.00009 | 100 |
| 8 | World Values Survey | I ‘d like to ask you how much you trust people from various groups. Could you tell me for each whether you trust people from this group completely, somewhat, not very much or not at all? People of another nationality | 1 = trust completely, 4 = do not trust at all | 1981-2018 (but varies by country) | 211855 | -0.002 | 0.00004 | 100 |
| 9 | World Values Survey | I ‘d like to ask you how much you trust people from various groups. Could you tell me for each whether you trust people from this group completely, somewhat, not very much or not at all? People you know personally | 1 = trust completely, 4 = do not trust at all | 1981-2018 (but varies by country) | 224599 | 0.008 | 0.001 | 100 |
| 10 | World Values Survey | And for which, if any, are you currently doing unpaid voluntary work? Unpaid work for human rights | [mentioned or not] | 1981-2018 (but varies by country) | 48958 | 0.0007 | 0.0004 | 100 |
| 11 | World Values Survey | Using the responses on this card, could you tell me how much you trust [Nationality] people in general? | 1 = trust completely, 4 = do not trust at all | 1981-2018 (but varies by country) | 21314 | 0.17 | 0.003 | 29.36 |
| 12 | World Values Survey | I ‘d like to ask you how much you trust people from various groups. Could you tell me for each whether you trust people from this group completely, somewhat, not very much or not at all? People you meet for the first time | 1 = trust completely, 4 = do not trust at all | 1981-2018 (but varies by country) | 221110 | -0.004 | 0.0001 | 100 |
| 13 | World Values Survey | I ‘d like to ask you how much you trust people from various groups. Could you tell me for each whether you trust people from this group completely, somewhat, not very much or not at all? Your family | 1 = trust completely, 4 = do not trust at all | 1981-2018 (but varies by country) | 22109 | -0.32 | 0.02 | 13.1 |
| 14 | World Values Survey | Generally speaking, would you say that most people can be trusted or that you need to be very careful in dealing with people? | 1 = most people can be trusted, 2 = need to be very careful | 1981-2018 (but varies by country) | 393873 | 0.003 | 0.002 | 100 |
| 15 | World Values Survey | Do you think most people would try to take advantage of you if they got a chance, or would they try to be fair? | 1 = most people try to take advantage of me, 10 = most people try to be fair | 1981-2018 (but varies by country) | 53007 | 0.02 | 0.0009 | 100 |
| 16 | European Social Survey | All rounds: Using this card, generally speaking, would you say that most people can be trusted, or that you can't be too careful in dealing with people? Please tell me on a score of 0 to 10, where 0 means you can't be too careful and 10 means that most people can be trusted. | 0 = you can't be too careful, 10 = most people can be trusted | 2002-2018 (but varies by country) | 421379 | 0.02 | 0.0002 | 100 |
| 17 | European Social Survey | Have you or a member of your household been the victim of a burglary or assault in the last 5 years? | 1 = yes, 2 = no | 2002-2018 (but varies by country) | 421416 | 0.004 | 0.00009 | 100 |
| 18 | European Social Survey | All rounds: Using this card, do you think that most people would try to take advantage of you if they got the chance, or would they try to be fair? | 0 = most people try to take advantage of me, 10 = most people try to be fair | 2002-2018 (but varies by country) | 418880 | 0.01 | 0.00008 | 100 |
| 19 | European Social Survey | Most of the time people helpful or mostly looking out for themselves | 0 = people mostly look out for themselves, 10 = people mostly try to be helpful | 2002-2018 (but varies by country) | 92774 | 0.04 | 0.0008 | 100 |
| 20 | European Social Survey | All rounds: How safe do you - or would you - feel walking alone in this area after dark? | 1 = all or most of the time, 2 = some of the time, 3 = just occasionally, 4 = never | 2002-2018 (but varies by country) | 418124 | -0.03 | 0.002 | 100 |
| 21 | European Social Survey | How often, if at all, do you worry about your home being burgled? | 1 = all or most of the time, 2 = some of the time, 3 = just occasionally, 4 = never | 2002-2018 (but varies by country) | 148663 | -0.01 | 0.00004 | 100 |
| 22 | European Social Survey | How often worry about becoming a victim of violent crime | 1 = all or most of the time, 2 = some of the time, 3 = just occasionally, 4 = never | 2002-2018 (but varies by country) | 148112 | -0.01 | 0.00002 | 100 |
| 23 | European Social Survey | Now I will briefly describe some people. Please listen to each description and tell me how much each person is or is not like you. Use this card for your answer. She/he thinks it is important that every person in the world should be treated equally. She/he believes everyone should have equal opportunities in life. | 1 = very much like me, 2 = like me, 3 = somewhat like me, 4 = a little like me, 5 = not like me, 6 = not like me at all | 2002-2018 (but varies by country) | 407185 | -0.005 | 0.00006 | 100 |
| 24 | European Social Survey | Now I will briefly describe some people. Please listen to each description and tell me how much each person is or is not like you. Use this card for your answer. It is important to her/him to listen to people who are different from her/him. Even when she/he disagrees with them, she/he still wants to understand them. | 1 = very much like me, 2 = like me, 3 = somewhat like me, 4 = a little like me, 5 = not like me, 6 = not like me at all | 2002-2018 (but varies by country) | 406259 | -0.02 | 0.0008 | 100 |
| 25 | European Social Survey | Now I will briefly describe some people. Please listen to each description and tell me how much each person is or is not like you. Use this card for your answer. It's very important to her/him to help the people around her/him. She/he wants to care for their well-being. | 1 = very much like me, 2 = like me, 3 = somewhat like me, 4 = a little like me, 5 = not like me, 6 = not like me at all | 2002-2018 (but varies by country) | 407386 | -0.04 | 0.003 | 100 |
| 26 | European Social Survey | Using this card, please tell me to what extent... ...you feel that people in your local area help one another? | 0 (not at all) - 6 (a great deal) | 2002-2018 (but varies by country) | 407185 | 0.05 | 0.001 | 100 |
| 27 | European Social Survey | Using this card, please tell me to what extent... ...you feel that people treat you with respect? | 0 (not at all) - 6 (a great deal) | 2002-2018 (but varies by country) | 93558 | -0.02 | 0.00004 | 100 |
| 28 | European Values Survey | Here is a list of qualities which children can be encouraged to learn at home. Which, if any, do you consider to be especially important? Please choose up to five. Tolerance and respect for other people | [participants could choose the quality from the list or not] | 1981-2017 (but varies by country) | 180972 | 0.004 | 0.006 | 100 |
| 29 | European Values Survey | Here is a list of qualities which children can be encouraged to learn at home. Which, if any, do you consider to be especially important? Please choose up to five. Unselfishness | [participants could choose the quality from the list or not] | 1981-2017 (but varies by country) | 179125 | 0.003 | 0.003 | 100 |
| 30 | European Values Survey | Please look carefully at the following list of voluntary organisations and activities and say ... b) which, if any, are you currently doing unpaid voluntary work for? Social welfare services for elderly, handicapped or deprived people | [participants could indicate they currently do unpaid work, or not] | 1981-2017 (but varies by country) | 122756 | -0.0002 | 0.00007 | 100 |
| 31 | European Values Survey | Please look carefully at the following list of voluntary organisations and activities and say ... b) which, if any, are you currently doing unpaid voluntary work for? And do you currently do any unpaid work for any of them? Third world development or human rights | [participants could indicate they currently do unpaid work, or not] | 1981-2017 (but varies by country) | 122661 | 0.005 | 0.001 | 100 |
| 32 | European Values Survey | Generally speaking, would you say that most people can be trusted or that you can't be too careful in dealing with people? | Most people can be trusted (recoded as 1), can't be too careful (recoded as 0) | 1981-2017 (but varies by country) | 178714 | 0.002 | 0.001 | 100 |
| 33 | European Values Survey | Here are some aspects of a job that people say are important. Please look at them and tell me which ones you personally think are important in a job? A useful job for society | [participants could choose the quality from the list or not] | 1981-2017 (but varies by country) | 163278 | -0.0002 | 0.00001 | 100 |

Additional Analyses in Study 5a

What explained participants’ overall perception of moral decline: their theories about personal change and interpersonal replacement within their personal worlds, or without? To find out, we fit three linear models on subsets of participants depending on the amount of information that they provided. The results are shown in Table S5. For each subset of participants, both personal change and interpersonal replacement for people in general were strong predictors of how much overall moral decline they perceived. For those participants who experienced personal change in their personal world, that change was not related to their overall perception of moral decline. For those participants who experienced both personal change and interpersonal replacement in their personal world, personal change was related to their overall perception of moral decline.

Table S5: Regression Coefficients for Each of the Four Models Fit in Study 5a

|  |  | **Personal change for people in general** | **Interpersonal replacement for people in general** | **Personal change for people in personal world** | **Interpersonal replacement for people in personal world** |
| --- | --- | --- | --- | --- | --- |
| **Subset** | **N** |  |  |  |  |
| Did not experience personal change or interpersonal replacement in personal world | 27 | *b* = 0.49  *t*(24) = 4.10  *p* < .001 | *b* = 0.42  *t*(24) = 3.75  *p* < .001 | -- | -- |
| Experienced personal change in personal world | 256 | *b* = 0.50  *t*(252) = 10.04  *p* < .001 | *b* = 0.31  *t*(252) = 8.72  *p* < .001 | *b* = 0.06  *t*(252) = 1.15  *p* = .25 | -- |
| Experienced both personal change and interpersonal replacement in personal world | 141 | *b* = 0.44  *t*(136) = 6.64  *p* < .001 | *b* = 0.30  *t*(136) = 5.46  *p* < .001 | *b* = 0.15  *t*(136) = 1.88  *p* = .061 | *b* = 0.08  *t*(136) = 1.02  *p* = .31 |

Note: Four participants provided an interpersonal replacement score for their personal world but not a personal change score. All tests are two-sided. No adjustments for multiple comparisons were applied because each model uses independent data.

Table S6: Demographics Questions Used in Studies 2a-c, 3, and 5a-b

| **Variable** | **Question** | **Response options** |
| --- | --- | --- |
| Age | What is your age? *(Please enter a whole number.)* | [Participants entered a whole number] |
| Gender | What is your gender? | Male, female, other |
| Race | What is your race/ethnicity? | American Indian or Alaska Native  Asian  Black or African-American  Hispanic or Latino Origin  Hawaiian or Pacific Islander  White  Other  More than 1 of the above |
| Education | What is the highest level of education you have completed? | Did not complete high school  High school diploma  Some college  Associate’s degree  Four-year college degree  Some graduate school  Graduate school |
| Number of children | How many children do you have? | 0, 1, 2, 3, 4+ |
| Political ideology | How would you describe yourself politically? | Very liberal  Somewhat liberal  Neither liberal nor conservative  Somewhat conservative  Very conservative |

Section 2: Additional Studies Not Appearing in Main Text

Study S1: Pre-registered Replication of Study 2a

Study S1 was a pre-registered replication of Study 2a. The preregistration is available at <https://osf.io/hr7we>.

Method

*Participants*. In November, 2022, we recruited a nationally representative sample of American adults using Prolific, an online sample provider. This sample was constructed to represent the American adult population in terms of gender, race, and age. We intended to recruit 1,000 participants, but Prolific ultimately provided us with 997 (512 female, 477 male, 8 “other”, M_age_ = 45.42, 76% white, 13% Black, 6% Asian, 4% Hispanic, 1% American Indian or Alaska Native, 1% “more than 1 of the above”). All participants were paid $0.75 each for their participation.

*Procedure*. Participants completed the same procedure as in Study 2a.

Results

*Exclusions.* One hundred and forty-three participants failed the attention check embedded in the demographics questions. An additional 113 participants failed the consistency check. As in Study 2a, and is specified in our preregistration plan, these participants were excluded from all analyses. As in Study 2a, these exclusions did not meaningfully affect any of the results.

*Main analysis.* As in Study 2a, we fit a mixed linear effects model with perceived morality as the outcome and a fixed effect of year and with random intercepts for each participant. We then conducted pairwise comparisons between each of the three years using a Holm-Bonferroni correction for multiple comparisons. The results of Study 2a were replicated: Participants rated people as less moral in 2022 (M = 4.25) than in both 2012 (M = 4.81, *b* = -0.56, *t*(1480) = -15.25, 95% CI = [-0.65, -0.48]) and 2002 (M = 5.06, *b* = -0.82, *t*(1480) = -22.03, 95% CI = [-0.90, -0.73]). Participants also rated people as less moral in 2012 than in 2002 (*b* = -0.25, *t*(1480) = -6.79, 95% CI = [-0.34, -0.16]).

Study S2: Replication of Study 2a with Participants from MTurk

Study 2a was run with participants recruited on the Prolific platform. Prolific provides nationally representative samples but is expensive and has a relatively small pool. In Study S2, we used the same method used in Study 2a with participants recruited on the MTurk platform, which is less expensive and has a larger pool. We reasoned that if the two platforms provided the same results, we could safely use the MTurk platform in subsequent studies.

Method

*Participants.* In January, 2020, 302 people responded to an advertisement for a study posted on Amazon Mechanical Turk. Respondents first took a three-item test of English language and American culture that required them to know that children in kindergarten are three or four years old, that an American ZIP code is a series of five digits, and that eating turkey is not an activity associated with Halloween. One hundred and two respondents failed to answer one or more of the questions correctly and were not allowed to participate. The remaining 200 respondents became participants in the study in exchange for $0.75 (68 female, 131 male, 1 “other”, M_age_ = 37.45 years, 60% White, 28% Black, 6% Hispanic, 4% Asian, 1% “more than one of the above”).

*Procedure.* Participants completed the same procedure as in Studies 2a and S1 with one additional question: After rating people in general today, “ten years ago,” and “twenty years ago,” they also rated people in general “thirty years ago.”

Results

*Exclusions.* Thirteen participants failed the attention check embedded in the demographics and were excluded from all analyses. Another participant reported that they are 300 years old and was excluded from all analyses. Finally, 53 participants gave answers to the consistency check question that were inconsistent with their previous answers; they were also excluded. This left 134 participants in all analyses (45 female, 88 male, 1 “other”, M_age_ = 38.09, 70% White, 19% Black, 5% Hispanic, 4% Asian, 1% “more than one of the above”, 1% “other”). These exclusions do not meaningfully affect the results.

*Main results.* Using the same analysis as in Studies 2a and S1, participants rated people in 2020 (M = 4.75) as less kind, honest, nice, and good compared to all previous years (2010: *b* = -0.26, 95% CI = [-0.51, -0.009], *t*(399) = -2.75, *p* = .02; 2000: *b* = -0.57, 95% CI = [-0.83, -0.32], *t*(399) = -6.05, *p* <.001; ,1990: *b* = -0.50, 95% CI = [-0.75, -0.25], *t*(399) = -5.27; *p* < .001). Participants rated people in 2010 (M = 5.01) as less kind, honest, nice, and good compared to all previous years (2000: *b* = 0.31, 95% CI = [-0.57, -0.06], *t*(399) = -3.30, *p* = .004; 1990: *b* -0.24, 95% CI = [-0.49, 0.013], *t*(399) = -2.51, *p* = .02). The difference in ratings for 2000 (M = 5.33) and 1990 (M = 5.25) was not significant, *b* = 0.07, 95% CI = [-0.18, 0.33], *t*(399) = 0.79, *p* = .43.

Study S3: Estimated Rates of Cooperation in Prisoner’s Dilemma Games (1956-2017)

A recent meta-analysis by Yuan et al (2022)^1^ found that between 1956 and 2017, cooperation rates in economic games––specifically, in prisoners’ dilemmas and public goods games––increased by 9% to 10% (depending on model specifications). In Study S3 (run in September, 2022), we described to participants a representative version of one of these games (based on Yi & Rachlin, 2004^2^, one of the studies included in the meta-analysis) and asked them to estimate whether and how cooperation rates might have changed over that time period.

Method

*Participants*. We recruited a sample of American adults using Prolific, an online sample provider. One hundred and forty-nine people (87 female, 60 male, 2 “other”, M_age_ = 35.19 years, 73% White, 7% Black, 6% Hispanic, 5% Asian, 1% Hawaiian or Pacific Islander, 3% other, 5% “more than one of the above”) were paid $1.00 each for their participation.

*Procedure*. After providing informed consent, participants confirmed their Prolific ID, per the site’s usage policy. They then completed the same three-item test of English language and American culture used in our other studies. Prolific does not allow participants to be screened out once they have begun the study, so if participants failed any of these questions, they were excluded later rather than being deemed ineligible before responding.

Participants then read and signed a consent form and reported their birth year, which was later compared to their self-reported age as a consistency check. Participants then read the following instructions: “For decades, researchers have been measuring how kind, nice, honest, and good people are by asking them to play a ‘cooperation game.’ Today we will explain the game and then ask you to guess what people did. If you guess correctly, we will automatically enter you in a lottery to win $20. Here is how one of the games worked. Two strangers reported to a laboratory and were put into separate rooms. They each sat at a computer and read the following instructions. Please read them closely because we'll ask you about them later.”

Participants then read a generic description of the Prisoner’s Dilemma game, based on Yi and Rachlin (2004)**^2^**, which was one of the studies included in the meta-analysis by Yuan et al (2022)^1^. “Today, you and the other person will each independently choose TO COOPERATE or NOT TO COOPERATE. If you choose TO COOPERATE, you will receive either $0 or $70. You will receive $0 if the other person chooses NOT TO COOPERATE, and they will receive $100. You will receive $70 if the other person chooses TO COOPERATE, and they will also receive $70. If you choose NOT TO COOPERATE, you will receive either $30 or $100. You will receive $30 if the other person chooses NOT TO COOPERATE, and they will receive $30. You will receive $100 if the other person chooses TO COOPERATE, and they will receive $0. So what would you like to do? Do you choose TO COOPERATE or NOT TO COOPERATE?”

On the next page, participants read: “As we already mentioned, researchers have been measuring how kind, nice, honest, and good people are by asking them to play this “cooperation game” for decades, and we now know the percentage of people who chose to cooperate in every year from 1956 to 2017. Do you think the rate of cooperation changed in that 61-year period, and if so, how?”

Participants responded by indicating one of three alternatives: (a) “I think the percentage of people who chose to cooperate went up between 1956 and 2017”; (b) “I think the percentage of people who chose to cooperate stayed the same between 1956 and 2017”; or (c) “I think the percentage of people who chose to cooperate went down between 1956 and 2017”.

Next, participants answered two questions by entering a number between 0 and 100 into a text box: (a) “In 1956, what percentage of people do you think chose to cooperate?” and (b) “In 2017, what percentage of people do you think chose to cooperate?”

Participants then completed two comprehension checks, which required them to demonstrate that they knew that (a) if one person chose to cooperate in the economic game and the other chose not to cooperate, the person who chose to cooperate got nothing, and (b) if both chose not to cooperate, they both got $30.

Finally, participants completed a set of demographics questions, embedded in which was another attention check that required participants to select the option “other” and type the word “shoe.”

Results

*Exclusions.* Three participants failed the language and culture test. An additional 30 participants failed at least one of the two comprehension checks. Another 12 failed the attention check embedded in the demographics questions. Another three participants reported an age at the end of the study that was inconsistent with the birth year they reported at the beginning of the study. Finally, seven participants gave inconsistent answers on the categorical and continuous questions (for instance, reporting that cooperation rates had increased, then giving the same answer for both continuous questions). These participants were excluded from all analyses, leaving 94 participants in the data set (54 female, 39 male, 1 “other”, M_age_ = 35.13, 69% White, 9% Hispanic, 7% Black, 6% Asian, 2% “other”, 5% “more than one of the above”). These exclusions did not meaningfully affect the results.

*Estimates of changes in cooperation rates.* When asked whether cooperation rates had increased, stayed the same, or decreased between 1956 and 2017, 26% of participants correctly reported that they had increased—but more than twice as many, 63%, mistakenly reported that they had decreased. Twelve percent of participants reported that they had stayed the same. This distribution of responses differed significantly from a distribution of response in which participants chose each option with equal frequency, χ^2^ (2, 94) = 39.34, *p* < .001. In addition, on average, participants estimated that in 1956, 63% of people cooperated and that in 2017 only 52% of people cooperate. A two-tailed paired-samples *t*-test indicated this difference was significant, *t*(93) = -5.01, 95% CI = [-15.02, -6.49], *p* < .001.

Section 3: Discussion of Related Literature

Other Demonstrations of the Perception of Moral Decline

The claim that people believe morality is declining is by no means new. Historians such as Arthur Herman^3^ and political scientists such as Andrew Murphy^4^ have traced the long history of belief in “moral declinism.” Psychologists Richard Eibach and Lisa Libby^5^ were (to our knowledge) the first to bring modern survey data to bear on this idea, describing a half dozen survey items on which people perceived moral decline to illustrate their suggestion that “Perceptions of moral decline are particularly common” and “often exaggerated.” In short, there is a long tradition of thought and a short tradition of research on the perception of moral decline and its potentially illusory status. However, to our knowledge, no one has ever *tested* the hypothesis that people perceive moral decline by analyzing the full corpus of survey data, as we did in Study 1. A recent study shows why testing the full corpus is imperative.

Mitchell and Tetlock^6^ asked participants (among other things) to guess how a variety of “social indicators” had changed over various time intervals. Most of these indicators had nothing to do with morality (e.g., dental health, hourly wages, educational attainment, etc.), but two were morally relevant: (a) the number of documented hate crimes committed on college campuses between 2015 and 2018 and (b) the percentage of youth in juvenile justice facilities who reported being sexually victimized between 2012 and 2018. Mitchell and Tetlock hypothesized that although the frequency of hate crimes and sexual victimization had decreased over the intervals and in the locations they specified, participants would mistakenly guess that these frequencies had increased. In other words, they expected participants to show an illusion of moral decline on these two items. Consistent with our results, that’s exactly what they found.

Although this evidence may have been satisfactory for Mitchell and Tetlock’s purposes, it does not provide satisfactory evidence of the illusion of moral decline because when researchers select just a few specific items for analysis (rather than analyzing the entire corpus of items), they can demonstrate anything they wish. For example, Mitchell and Tetlock asked participants to estimate the frequency of hate crimes that had occurred *between 2015 and 2018* and *on college campuses*. Figure S5 shows the original data on which Mitchell and Tetlock report having based their question. Note first that across the full interval (from 2010 to 2019), there is no meaningful linear trend: The correlation between year and frequency of hate crimes on college campuses in these data is *r* = .1. From these data that show no linear trend over 10 years, Mitchell and Tetlock selected a specific 3-year interval (2015 to 2018) in which hate crimes on college campuses happen to have decreased by 3%.


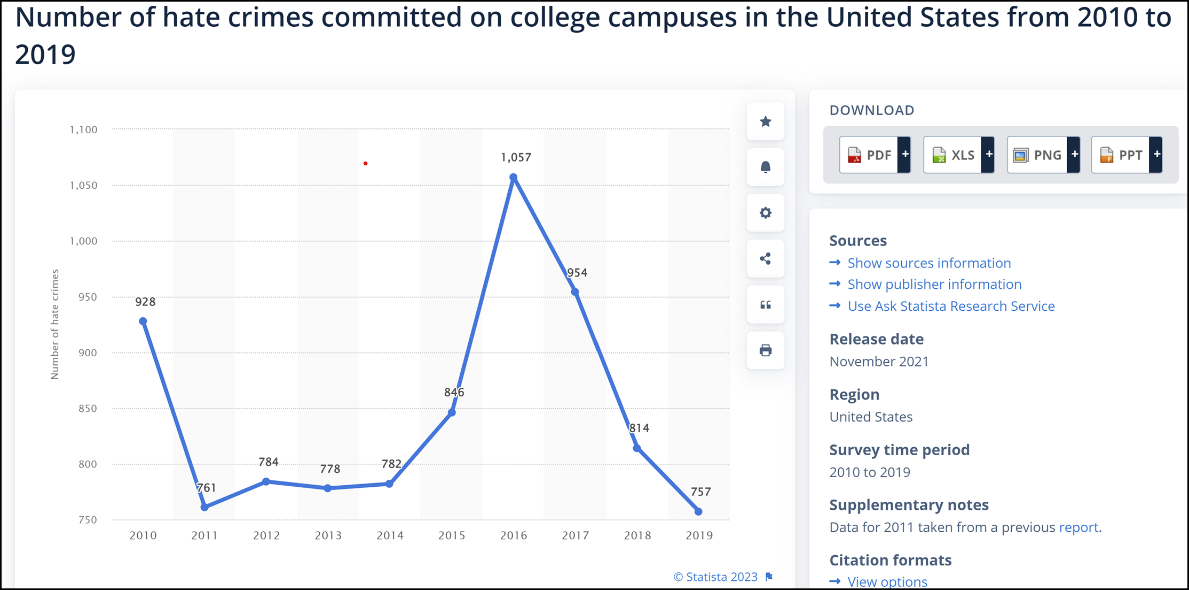


**Figure S5. Data on which Mitchell and Tetlock (2022) based their question about the frequency of hate crimes on college campuses between 2013 and 2016.**

Mitchell and Tetlock chose a special interval and also a special location: college campuses. Figure S6 shows the frequency of hate crimes in the U.S. in the previous decade according to the FBI’s Uniform Crime Reporting Hate Crime Statistics Program (available at <https://bjs.ojp.gov/library/publications/hate-crime-recorded-law-enforcement-2010-2019>). Although the rate of hate crimes that occurred *on college campuses* decreased by 3% between 2015 and 2018, the rate of hate crimes that *occurred in the U.S.* increased by 22% in that same interval. In other words, Mitchell and Tetlock selected a specific interval and a special location in which there was a slight decrease in hate crimes, in contrast with the more general trend. A participant who was (correctly) aware that the frequency of U.S hate crimes had increased in general might naturally (but incorrectly) have guessed that it also increased in this specific location during this specific interval.


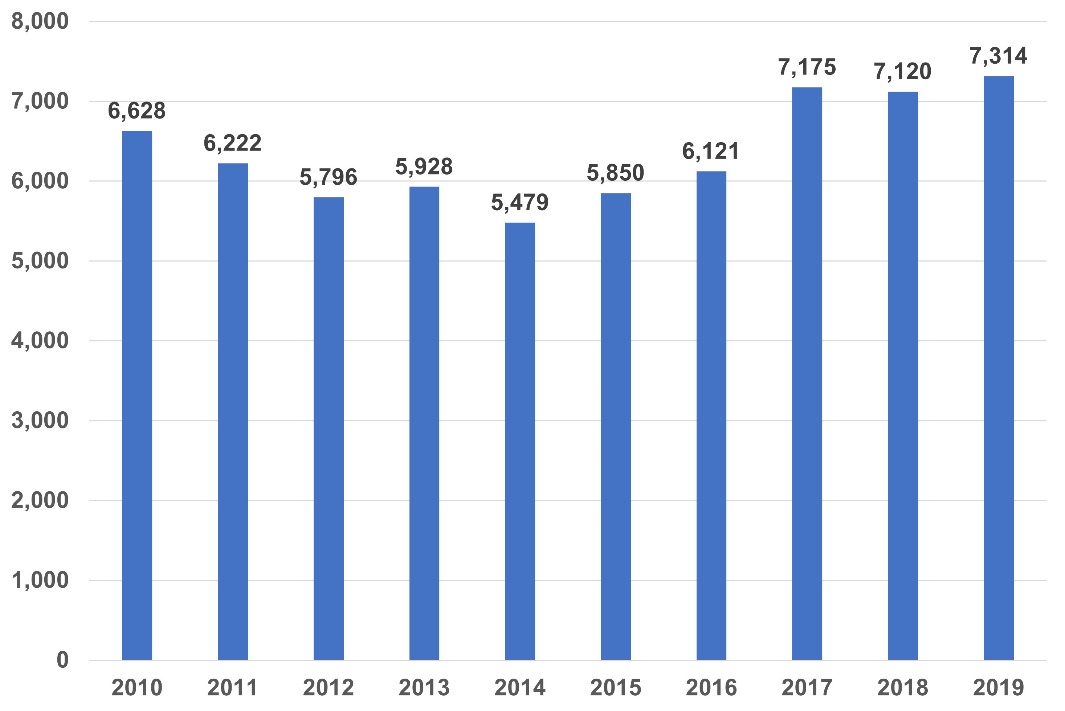


**Figure S6. Frequency of hate crimes in the US between 2010 and 2019.**

The point here is that producing an illusion of moral decline, an illusion of moral improvement, or no illusion at all, is not difficult when researchers design or select a small number of items to analyze, which is why previous analyses of a small number of handpicked items do not provide a meaningful test of the hypothesis that the perception of moral decline is “common” and “exaggerated.” Our Studies 1 and 4, in contrast, examine all the relevant indicators we could find from all the major survey providers in all the relevant time intervals over three quarters of a century, and as such, they are (to our knowledge) the first to test this hypothesis.

Potential Causes of the Perception of Moral Decline

Previous research has identified several phenomena that could play a role in producing the illusion of moral decline.

- Eibach and colleagues^5,7–9^ have shown that ordinary life transitions—such as the transition to parenthood—can make people more aware of external threats, and that people may then mistake the changes in their levels of subjective concern for changes in the levels of objective threat. For example, because people start to worry more about dangers in the world after having children, parents may mistakenly conclude that the world itself has become more worrisome. It should be clear how this important phenomenon can lead to an illusion of moral decline. However, as far as we can tell, it does not play a consistent role in the illusion of moral decline documented by our studies. In Study 5a, parents perceived *less* moral decline than non-parents when all other demographic factors were accounted for. In Study 3, parents perceived *more* moral decline than non-parents. (These exploratory models also accounted for race, gender, age, education, and political orientation.) In the rest of our studies, parental status did not predict the perception of moral decline. The effect described by Eibach and colleagues is intuitively compelling, but we could not find consistent evidence that it played a role in our studies. The absence of evidence is not, of course, evidence of absence, and it is possible that the effects of parental status are specific to perceiving increases in danger rather than perceiving decreases in general morality, or that the effects were simply “swamped” by the noise created by the heterogeneity of the items we analyzed.
- Protzko and Schooler^10,11^ have shown that older people tend to derogate younger people in part because older people mistakenly remember being “better youngsters” than the ones they see around them. The "kids these days” effect may help explain why participants in Study 3 attributed some portion of moral decline to “interpersonal replacement” (which is the belief that newer generations are less moral than older generations). Of course, participants in Study 3 also attributed some portion of moral decline to “personal change” (which is the belief that individuals become less moral as they move through time), which suggests that while the “kids these days” effect is an important source of the illusion of moral decline documented in our studies, it is not the sole source. It is also worth noting that even the youngest participants in our studies perceived moral decline, and that the amount of moral decline perceived by participants in Studies 2c and 5b was unrelated to their ages once we controlled for the amount of time about which participants were being asked to make judgments.
- Stavrova and Ehlebracht have shown that people tend to equate cynicism with cognitive ability ^12^, which suggests that people’s reports of moral decline could simply be attempts to impress others with their intelligence and perspicacity. Although cynicism may well be a winning impression-management strategy in many instances, we do not believe it plays a significant role in the illusion of moral decline documented by our studies. First, participants in our original studies responded anonymously to questions on an online survey, so there was no “other person” whose impressions they might manage. Anonymous responding was likely the case for many if not most of the archival surveys we analyzed in Study 1. Second, participants in both the archival studies and our original studies were willing to report increases in morality on certain dimensions (e.g., the treatment of African Americans, people with physical disabilities, and gay people) and among certain populations (e.g., people in their personal worlds), which is not what one would expect of people who were attempting to appear cynical. Third, when we gave participants a financial incentive to accurately predict the direction of change in rates of cooperation in the prisoner’s dilemma game over 61-years (see Study S3 in Section 3), a substantial majority mistakenly estimated that the rate had declined when, in fact, it has increased. Although it is always difficult to know whether people’s responses to survey questions were influenced by their concerns with other people’s the impressions of them, we see little reason to suspect that such concerns played a role in the illusion of moral decline documented by our studies.

We would note also that the BEAM mechanism made specific predictions (i.e., that people believe that moral decline began about the time they were born and do not believe it occurred among people in their personal worlds) that were confirmed in Studies 5a and 5b, and that none of the foregoing phenomena easily explain.

Section 4: Mathematical Model of the BEAM Mechanism

We suggest that biased exposure (i.e., people encounter and attend to more negative moral information than positive moral information) and biased memory (i.e., the impact of the negative moral information in memory diminishes more quickly than does the impact of the positive moral information in memory) can together produce the illusion of moral decline. We refer to this “Biased Exposure And Memory” as the “BEAM mechanism.” To show how the mechanism works, we offer a mathematical model:

*M = min[n_n_(A_n_ + t*r_n_), 0] + max[n_p_(A_p_ - t*r_p_), 0]*

…where *M* is the observer’s perception of the morality of “people in general” at a given point in time *t* (expressed in years, for convenience); *n_n_* is the number of units of negative moral information the observer encountered at that time *t*; *n_p_* is the number of units of positive moral information the observer encountered at that time *t*; *A_n_* is the average negativity of the negative information encountered at that time *t* expressed as a negative number; *A_p_* is the average positivity of the positive information encountered at that time *t* expressed as a positive number; *r_p_* is the rate at which the impact of the positive information decreases in memory over time; and *r_n_* is the rate at which the impact of negative information decreases in memory over time. The *max* and *min* functions and the trailing zeroes merely prevent the overall amount of negativity or positivity from crossing zero. This is because the impact of positive and negative information may decrease to zero, but positive information does not tend to become negative nor does negative information tend to become positive, on average. This model is simply a formal statement of the claim that an observer’s perception of the morality of people in general at a particular time is a function of the information to which the observer was exposed at that time and the emotional impact that information currently has on the observer.

What does the model predict when both exposure and memory are biased in the ways that previous research suggests they actually are? To model the effects of biased exposure, we let *n_p_* < *n_n_*, and to model the effects of biased memory, we let *r_p_* < *r_n_* . For example, we can let *n_p_* = 5 and *n_n_* = 15, which means that the observer encounters three times as much negative moral information as positive moral information. We can also let *r_p_* = 0.1 and *r_n_* = 0.2, which means that with each passing year, the positive moral information loses .1 units of impact and the negative moral information loses .2 units of impact. Finally, we can let *A_p_* = 5 and *A_n_* = -5, which means that the positivity of the positive moral information is equivalent to the negativity of the negative moral information.

The left panel of Figure ED2 shows the predictions of the model under these assumptions. Perceived morality is plotted on the y-axis and time is plotted on the x-axis such that T = 0 is the present, and T – *x* is *x* years in the past. The black circles show the observer’s perception of current morality, which is the perception of morality at each value of T by an observer who is also at that value of T. The grey circles show the perception of past morality, which is the perception of morality at each value of T by an observer who is at T = 0. As the figure shows, under the assumptions of the model, observers will perceive a low and equally low level of “current morality” at all time points (because of biased exposure) but will remember “past morality” as high—and higher with each additional year that separates the present and the past (because of biased memory). In other words, if we assume that both exposure and memory are biased in the ways that previous research suggests they actually are, the BEAM mechanism predicts that observers will experience an illusion of moral decline.

The BEAM mechanism also predicts that when people are exposed to a disproportionate amount of *positive* information about morality rather than a disproportionate amount of negative information (as they are with their family, friends, and close associates), they should perceive moral improvement rather than moral decline, which is what we found in Study 5a. Specifically, let us assume that at every point in time an observer is exposed to more positive moral information than negative moral information (i.e., let *n_p_* = 15 and *n_n_* = 5) and let us retain all previous assumptions. If we do this, then (as the center panel of Figure ED2 shows) the model predicts that the observer will perceive a high and equally high level of “current morality” at all time points (because of biased exposure) but will remember “past morality” as low—and lower with each additional year that separates the present and the past (because of biased memory).

Additionally, as the right panel of Figure ED2 shows, the BEAM mechanism predicts that people should not perceive moral decline in periods for which they have no information in memory, which is to say that biased exposure by itself cannot account for the perception of moral decline.

**
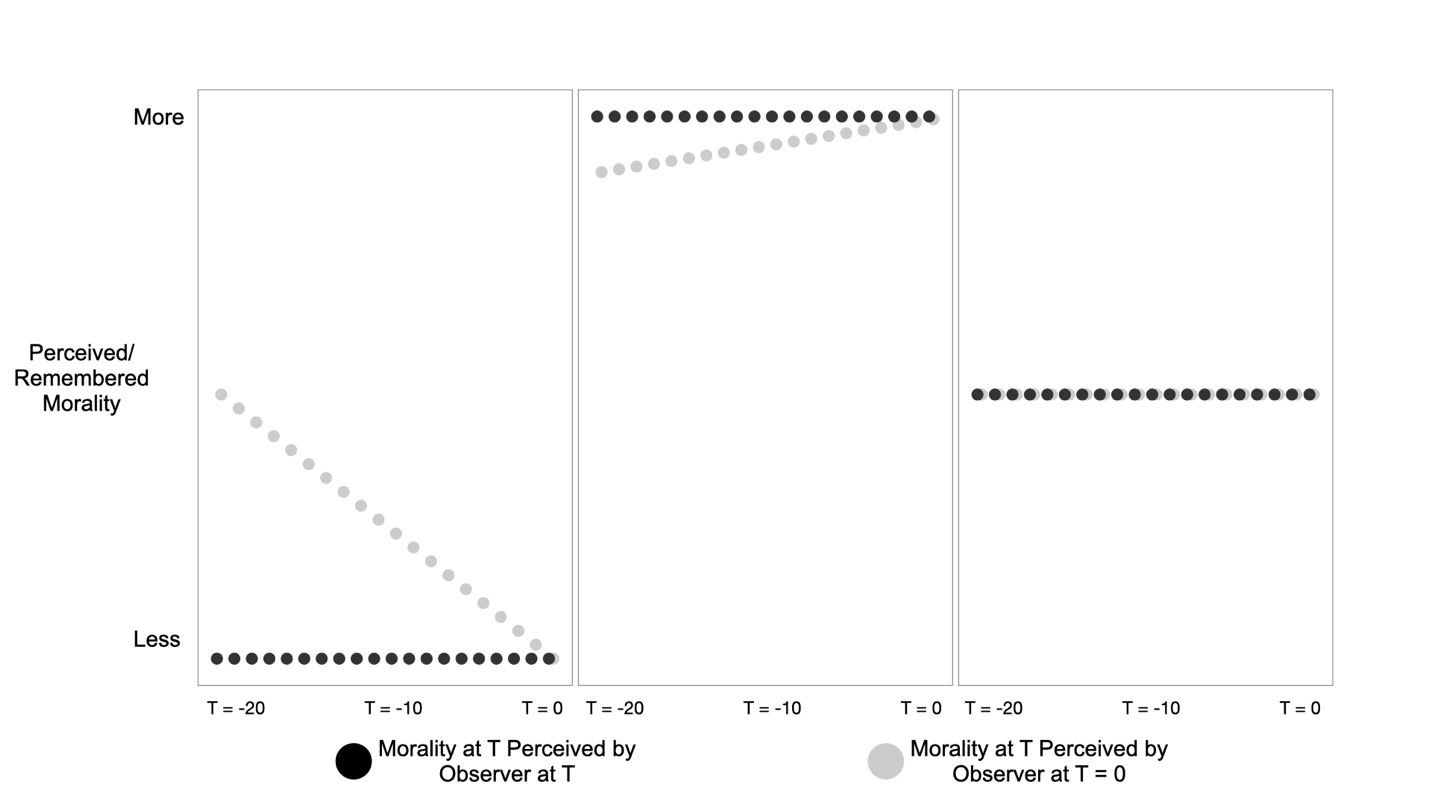
**

**Figure S1.** Data generated using the BEAM model. Left panel: Simulated data generated by setting *n_p_* = 5, *n_n_* = 15, *A_p_* = 5, *A_n_* = -5, *r_p_* = 0.1, and *r_n_* = 0.2. Center panel: Simulated data generated by setting *N_p_* = 15, *N_n_* = 5, *A_p_* = 5, *A_n_* = -5, *r_p_* = 0.1, and *r_n_* = 0.2. Right panel: Simulated data generated by setting *N_p_* = 0, *N_n_* = 0, *A_p_* = 0, *A_n_* = 0, *r_p_* = 0.1, and *r_n_* = 0.2. Points are dodged slightly to show all data.

Section 5: References in Supplemental Information Section

1. Yuan, M. *et al.* Did cooperation among strangers decline in the United States? A cross-temporal meta-analysis of social dilemmas (1956–2017). *Psychol. Bull.* **148**, 129–157 (2022).

2. Yi, R. & Rachlin, H. Contingencies of reinforcement in a five-person prisoner’s dilemma. *Journal of the Experimental Analysis of Behavior* vol. 82 161–176 (2004).

3. Herman, A. *The idea of decline in western history*. (Free Press, 1997).

4. Murphy, A. R. Augustine and the Rhetoric of Roman Decline. *Hist. Polit. Thought* **26**, 586–606 (2005).

5. Eibach, R. P. & Libby, L. K. Ideology of the good old days: Exaggerated perceptions of moral decline and conservative politics. in *Social and psychological bases of ideology and system justification* (Oxford University Press, 2009).

6. Mitchell, G. & Tetlock, P. E. Are progressives in denial about progress? Yes, but so is almost everyone else. *Clin. Psychol. Sci.* 21677026221114316 (2022) doi:10.1177/21677026221114315.

7. Eibach, R. P., Libby, L. K. & Ehrlinger, J. Unrecognized changes in the self contribute to exaggerated judgments of external decline. *Basic Appl. Soc. Psych.* **34**, 193–203 (2012).

8. Eibach, R. P. & Mock, S. E. The vigilant parent: Parental role salience affects parents’ risk perceptions, risk-aversion, and trust in strangers. *J. Exp. Soc. Psychol.* **47**, 694–697 (2011).

9. Eibach, R. P., Libby, L. K. & Gilovich, T. D. When change in the self is mistaken for change in the world. *J. Pers. Soc. Psychol.* **84**, 917–931 (2003).

10. Protzko, J. & Schooler, J. W. Who denigrates today’s youth?: The role of age, implicit theories, and sharing the same negative trait. *Frontiers in Psychology* vol. 13 (2022).

11. Protzko, J. & Schooler, J. W. Kids these days: Why the youth of today seem lacking. *Sci. Adv.* **5**, eaav5916 (2022).

12. Stavrova, O. & Ehlebracht, D. The cynical genius illusion: Exploring and debunking lay beliefs about cynicism and competence. *Personal. Soc. Psychol. Bull.* **45**, 254–269 (2018).
