# Supplementary material for: The illusion of moral decline
Source: Nature. 2023 Jun 7;618(7966):782–9. doi: 10.1038/s41586-023-06137-x (PMC10284688; doi:10.1038/s41586-023-06137-x)
Supplement: Supplementary file 2 — Reporting Summary [file 41586_2023_6137_MOESM2_ESM.pdf]

nature portfolio

Corresponding author(s): Adam Mastroianni

Last updated by author(s): Mar 8, 2023

Reporting Summary

Nature Portfolio wishes to improve the reproducibility of the work that we publish. This form provides structure for consistency and transparency in reporting. For further information on Nature Portfolio policies, see our Editorial Policies and the Editorial Policy Checklist.

Statistics

For all statistical analyses, confirm that the following items are present in the figure legend, table legend, main text, or Methods section.

n/a	Confirmed	
☐	☒	The exact sample size (*n*) for each experimental group/condition, given as a discrete number and unit of measurement
☐	☒	A statement on whether measurements were taken from distinct samples or whether the same sample was measured repeatedly
☐	☒	The statistical test(s) used AND whether they are one- or two-sided<br>*Only common tests should be described solely by name; describe more complex techniques in the Methods section.*
☐	☒	A description of all covariates tested
☐	☒	A description of any assumptions or corrections, such as tests of normality and adjustment for multiple comparisons
☐	☒	A full description of the statistical parameters including central tendency (e.g. means) or other basic estimates (e.g. regression coefficient) AND variation (e.g. standard deviation) or associated estimates of uncertainty (e.g. confidence intervals)
☐	☒	For null hypothesis testing, the test statistic (e.g. *F*, *t*, *r*) with confidence intervals, effect sizes, degrees of freedom and *P* value noted<br>*Give P values as exact values whenever suitable.*
☐	☒	For Bayesian analysis, information on the choice of priors and Markov chain Monte Carlo settings
☐	☒	For hierarchical and complex designs, identification of the appropriate level for tests and full reporting of outcomes
☐	☒	Estimates of effect sizes (e.g. Cohen's *d*, Pearson's *r*), indicating how they were calculated

Our web collection on statistics for biologists contains articles on many of the points above.

Software and code

Policy information about availability of computer code

Data collection	Original data was collected using Qualtrics survey software.
Data analysis	Data analysis was conducted using the R statistical software version 4.0.2. Additionally, we used the following packages: lme4 (v. 1.1-23), lmerTest (3.1-2) and emmeans (1.4.7). All external packages used are included in the code package available on OSF: https://osf.io/t83zy/

For manuscripts utilizing custom algorithms or software that are central to the research but not yet described in published literature, software must be made available to editors and reviewers. We strongly encourage code deposition in a community repository (e.g. GitHub). See the Nature Portfolio guidelines for submitting code & software for further information.

Data

Policy information about availability of data

All manuscripts must include a data availability statement. This statement should provide the following information, where applicable:

- Accession codes, unique identifiers, or web links for publicly available datasets
- A description of any restrictions on data availability
- For clinical datasets or third party data, please ensure that the statement adheres to our policy

All original data is publicly available on OSF: https://osf.io/t83zy/. The archival data is proprietary and cannot be shared publicly, but the data documents for Studies 1 and 4 include links through which data can be accessed for anyone with institutional access.

Human research participants

Policy information about studies involving human research participants and Sex and Gender in Research.

Reporting on sex and gender	Participants self-reported their gender in our original studies by selecting "male", "female", or "other". We did not hypothesize any interactions between gender and our main results. We included gender as a covariate in our exploratory analyses, and no effects were significant in any study.
Population characteristics	Demographics questions are listed verbatim in Table S6 in the Supplementary Material. Demographic breakdowns for each study before and after exclusions are given in the Concluding Methods.
Recruitment	In our original studies, participants were recruited on the Prolific platform and on the Amazon Mechanical Turk platform via an advertisement for a short study.
Ethics oversight	All studies were approved by the Institutional Review Board of Harvard University.

Note that full information on the approval of the study protocol must also be provided in the manuscript.

Field-specific reporting

Please select the one below that is the best fit for your research. If you are not sure, read the appropriate sections before making your selection.

☐ Life sciences ☒ Behavioural & social sciences ☐ Ecological, evolutionary & environmental sciences

For a reference copy of the document with all sections, see nature.com/documents/nr-reporting-summary-flat.pdf

Behavioural & social sciences study design

All studies must disclose on these points even when the disclosure is negative.

Study description	Our studies are mixed methods. Studies 1 and 4 are analyses of archival data. Studies 2a-c, 3, 5a-b, and S1-S3 are original surveys.
Research sample	The samples for Studies 2a and S1 are from the Prolific survey platform and it is designed to be representative of the adult population of the United States in terms of age, race, and gender. The samples for Studies 2b, 3, and 5a, S2, and S3 are convenience samples from Amazon Mechanical Turk. Studies 2c and 5b are from Amazon Mechanical Turk, but were additionally filtered to get a broader range of age representation. These samples were used because previous research has suggested that they are broadly similar to other samples traditionally used in psychology (e.g., Paolacci, Chandler, Ipeirotis, 2010). Archival data for Studies 1 and 4 came from a large number of polling data providers, including Gallup, Pew, the American National Election Studies, and the General Social Survey, as well as hundreds of results indexed in the Roper iPoll online database.
Sampling strategy	The samples for Studies 2a and S1 were designed to be nationally representative of the adult population of the United States in terms of age, race, and gender by the Prolific platform. All other original studies were open to anyone on Amazon Mechanical Turk, with the exceptions described above. In our original studies, because we did not know the size of the effect we were seeking to detect, we used a guideline of the sample size required to detect an effect of d = .3 using a two-tailed, one-sample t-test (N = 146). We collected larger samples than this number for a number of reasons: a) to account for possible exclusions, b) to obtain a nationally representative sample (Study 2a) or samples with broader age representation (Studies 2c and 5b), and c) to ensure that we reached sufficient numbers of participants who experienced cohort replacement and individual change in their personal worlds (Study 5a). Sample sizes were determined a priori and no data was analyzed before reaching our target N.
Data collection	For studies 2a-c, 3, and 5a-b, and S1-3, participants completed the survey online at a time and place of their choosing. The researchers were not present.
Timing	Study 2a: June 2020<br>Study 2b: January 2020<br>Study 2c: January 2020<br>Study 3: March 2020<br>Study 5a: May 2020<br>Study 5b: March 2021<br>Study S1: November 2022<br>Study S2: January 2020<br>Study S3: September 2022<br><br>The archival data used in Studies 1 and 4 were collected between 1949 and 2020.
Data exclusions	Participants were only excluded if they failed one or more attention and quality control checks, which were specific to each study. These are listed for each study in the Concluding Methods. We also note whenever exclusions affect results. These exclusions were decided upon a priori.
Non-participation	We report for each study in the Concluding Methods whether there were any participants who began but did not finish the study.

Randomization	Participants were not randomized into conditions because all of our original studies were within-subjects surveys.

Reporting for specific materials, systems and methods

We require information from authors about some types of materials, experimental systems and methods used in many studies. Here, indicate whether each material, system or method listed is relevant to your study. If you are not sure if a list item applies to your research, read the appropriate section before selecting a response.

Materials & experimental systems

n/a	Involved in the study
☒	☐ Antibodies
☒	☐ Eukaryotic cell lines
☒	☐ Palaeontology and archaeology
☒	☐ Animals and other organisms
☒	☐ Clinical data
☒	☐ Dual use research of concern

Methods

n/a	Involved in the study
☒	☐ ChIP-seq
☒	☐ Flow cytometry
☒	☐ MRI-based neuroimaging
